# High-Risk Rhabdomyosarcomas Feature a Convergent Cell State

**DOI:** 10.1158/0008-5472.CAN-25-4403

**Published:** 2026-07-14

**Authors:** Holly J. Whitfield, Nathaniel D. Anderson, Christina Burke, Marian J. Groot Koerkamp, Conor Parks, Toochi Ogbonnah, Yvette Wood, Alice Piapi, Emilia Robertson, Eleanor Watt, Abigail White, Solange De Noon, Jonathan Kennedy, Rajvi Nagrecha, Michael T. Meister, Ewa Aladowicz, Yang Kee Stella Man, Virginia Laspidea, Mi K. Trinh, Angus Hodder, Tarryn Porter, John E. Lawrence, Elizabeth Tuck, Trung Nguyen, Anna Kelsey, Adrienne M. Flanagan, Richard Hewitt, Naima Smeulders, Olga Slater, J. Ciaran Hutchinson, Neil Sebire, Janet M. Shipley, Jarno Drost, Karin Straathof, Sam Behjati

**Affiliations:** 1 Wellcome Sanger Institute, Hinxton, United Kingdom.; 2 UCL Cancer Institute, London, United Kingdom.; 3 Princess Máxima Center for Pediatric Oncology, Oncode Institute, Utrecht, the Netherlands.; 4 Great Ormond Street Hospital for Children, London, United Kingdom.; 5 Royal National Orthopaedic Hospital, Middlesex, United Kingdom.; 6 Institute of Cancer Research, Sutton, United Kingdom.; 7 Royal Manchester Children’s Hospital, Manchester Foundation Trust, Manchester, United Kingdom.; 8 Division Cell Biology, Metabolism and Cancer, Department Biomolecular Health Sciences, Faculty of Veterinary Medicine, Utrecht University, Utrecht, the Netherlands.; 9 Cambridge University Hospitals NHS Foundation Trust, Cambridge, United Kingdom.; 10 Department of Paediatrics, University of Cambridge, Cambridge, United Kingdom.

## Abstract

Somatic mutations often predict survival in childhood cancers, as exemplified in rhabdomyosarcoma (RMS), in which *FOXO1* gene fusion status is a key prognostic feature. In this study, we examined single-cell transcriptomes and discovered that cancer cells of lethal disease converge on a common cell state with a shared transcriptional landscape, irrespective of fusion status. Nuclear transcriptomics, chromatin accessibility, spatial transcriptomics, perturbation studies, and previously published datasets validated the overarching high-risk cell state. The convergent cell state only partially overlapped with transcriptional effects of the *FOXO1* fusion and unexpectedly exhibited neural features. Overall, these findings delineate a cell state of high-risk RMS cells that transcends conventional molecular and histologic boundaries, suggesting an overarching disease phenotype that could transform target discovery and inform clinical practice.

**Significance:**

Defining a convergent cell state of high-risk rhabdomyosarcoma that transcends existing histological and molecular features predictive of outcome provides biological insights into aggressive disease and uncovers potential biomarkers and therapeutic targets.

## Introduction

Rhabdomyosarcoma (RMS) is a soft tissue cancer of childhood that is marked by abnormal skeletal muscle differentiation (1–3). A variety of parameters such as age, disease site, and tumor spread determine outcomes in RMS. The most impactful prognostic factor, however, is the presence of a somatic rearrangement involving the *FOXO1* gene, which is usually partnered with *PAX3* or *PAX7*. The resulting gene fusion operates as an oncogenic transcription factor (TF) that perturbs muscle cell development and confers a particularly poor prognosis (4, 5). Morphologically, most fusion-positive tumors display “alveolar” histology (resembling lung alveoli) whereas fusion-negative tumors are generally “embryonal” in appearance (akin to developing muscle cells; ref. 6). Accordingly, prior to the discovery of the *FOXO1* gene fusion, alveolar appearance had already been associated with poor outcomes (7). Given the profound effect of the presence of the fusion on survival, histology no longer features as an independent risk factor in current practice (provided fusion status can be determined).

A fundamental biological difference between fusion-positive and -negative tumors is the degree of muscle differentiation they display, which is a direct effect of the gene fusion. Cells harboring the fusion usually exhibit immunohistochemical (IHC) markers of mature skeletal muscle, whereas embryonal tumors resemble more primitive muscle cells (8, 9). Recent studies that examine the differentiation of RMS cells through single-cell transcriptomics have substantiated this difference between fusion-positive and -negative RMS cells (10–14). Collectively, these studies have demonstrated stark differences in the maturity of RMS cells relative to human fetal muscle cells that extend beyond the resemblance of fusion-positive cells to more mature cells. For instance, mesenchymal cell states have been described in embryonal, fusion-negative cancer cells.

When fusion-negative RMS is lethal, this may be explained by a variety of factors such as advanced disease at diagnosis, anatomic challenges of treating the primary lesion (e.g., anatomically inaccessible areas), or specific genetic features, such as the *MYOD1* L122R hotspot mutation (15). Given the profound effects of the *FOXO1* fusion on muscle differentiation, we hypothesized that a cancer cell state of aggressive disease may exist. We pursued this hypothesis in a cohort of children with RMS, using single-cell mRNA, nuclear RNA sequencing (RNA-seq) combined with an assay for transposase-accessible chromatin (ATAC), single-cell spatial readouts, and perturbation experiments.

## Materials and Methods

### Sample acquisition and patient characteristics

RMS tumors and matched blood samples were collected from Great Ormond Street Hospital in London, England, in accordance with the Institutional Research Ethical Board guidelines. Each specimen underwent extensive pathologic review by expert pathologists. Detailed clinical information (age at presentation, sex, tumor site, stage, etc.) was obtained. Outcome data as of September 10, 2024, were also collected. Overall, the patients’ clinical features and demographics were typical of RMS: the average age at diagnosis was 5.3 years (0–12 years); the male to female ratio was 3:1. Further information can be found in Supplementary Table S1.

### Ethics statement

This study was conducted in accordance with recognized ethical guidelines. Tissues were collected through studies approved by United Kingdom (UK) National Health Service (NHS) research ethics committees and conducted in accordance with applicable UK ethical and regulatory requirements for health research and human tissue research. This includes NHS Research Ethics Committee review and the Human Tissue Act 2004, where relevant. Patients or guardians provided informed written consent for participation in this study as stipulated by the study protocols. These studies have the following references: NHS National Research Ethics Service reference 16/LO/0960 and NHS National Research Ethics Service reference 16/EE/0394. Additional archival samples were provided by the VIVO Biobank (formerly Children’s Cancer and Leukemia Group) under NHS National Research Ethics Service reference 23/EM/0130.

### Tumor tissue processing and single-cell suspension preparation for 10x Chromium analysis

Surplus tumor tissue, obtained during diagnostic biopsy or surgical resection, was processed within 1 hour of retrieval in the histopathology laboratory. Upon arrival, tissue samples were finely chopped using a scalpel and incubated in RPMI 1640 medium supplemented with 20% fetal calf serum and 1% L-glutamine. Collagenase IV (1.6 mg/mL; cat. #11410982, MP Biomedicals) was added, and the mixture was maintained at 37°C for 30 minutes with inversion every 10 minutes. Following enzymatic digestion, the resulting material was filtered through a 70-μm cell strainer and subsequently subjected to red blood cell lysis using a 1× RBC lysis buffer (cat. #420301; BioLegend, RRID: SCR_001134) for 10 minutes at ambient temperature.

The resulting single-cell suspensions were used for further applications. In selected cases, enrichment for tumor cells was achieved by depleting CD45^+^ cells using the CD45 MicroBead Kit (cat. #130-045-801; Miltenyi Biotec, RRID: AB_2783001) in accordance with the manufacturer’s instructions. For samples undergoing CD45 depletion, both the depleted and nondepleted fractions were subsequently treated using the Dead Cell Removal Kit (cat. #130-090-101; Miltenyi Biotec), as per protocol, to eliminate nonviable cells. In instances in which CD45 depletion was not performed, full single-cell suspensions were processed directly. Final viable cell suspensions, including both CD45-depleted and nondepleted preparations, were loaded onto the 10x Genomics Chromium platform (RRID: SCR_019326) for downstream analyses.

### 10x library preparation and sequencing

Single-cell suspensions were manually counted using a hemocytometer and adjusted to a concentration of 1,000 cells/μL. Cells were then processed following the standard protocol of either the Chromium Single Cell 3′ Kit (v2 and v3 chemistry) or the Chromium Single Cell 5′ kit (R2-only). All subsequent steps were performed in accordance with the manufacturer’s instructions. Libraries from five to eight 10x chip positions (channels) were pooled and sequenced using one lane of a 150 bp S4 flow cell on the Illumina NovaSeq 6000 (RRID: SCR_016387).

### Alignment and processing of single-cell RNA-seq data

Single-cell RNA-seq (scRNA-seq) data were mapped to the GRCh38 (v1.2.0) human reference genome and quantified using cellranger (v8.0.0, RRID: SCR_017344). Low-quality cells were removed based on the number of genes detected per cell (>500) and % of reads mapping to mitochondrial genes (10%–15%), in which thresholds were adjusted based on sequencing quality. Additional quality control was performed for two samples—FN_MYOD1_2 and FN_OTHER_8—with uniquely poor quality. For FN_MYOD1_2, we found both an inflation in the number of likely doublets as well as many cells with poor gene count. To ensure doublet removal, we discarded cells with >8,000 genes and used a count-by-Unique Molecular Identifier (UMI) metric (# genes/total counts) to remove cells with a small number of genes. For FN_OTHER_8, we removed cells with >10% of reads mapping to ribosomal genes, which seemed to form a cluster of low-quality cells.

Raw count matrices were processed using scanpy (v1.11.3, RRID: SCR_018139; ref. 16), which included filtering genes that were expressed in fewer than 20 cells, counts per million (CPM) normalization, and log-transformation. Normalized counts were then used for principal component analysis (PCA), Uniform Manifold Approximation Projection (UMAP), and leiden clustering. Scrublet (v0.2.3, RRID: SCR_018098; ref. 17) was used for doublet detection, and all scoring, including for cell-cycle genes, was performed with scanpy. Cells were annotated based on the expression of canonical marker genes (Supplementary Fig. S1). Samples with no cancer cells were excluded from analyses. Two samples (diagnostic and refractory) from the same patient (FN_OTHER_7) were combined for analyses as the diagnostic sample contained no cancer cells.

### Multiome single-nuclear RNA and ATAC-seq sample preparation

Frozen tissue embedded in optimal cutting temperature (OCT) was sectioned at a thickness of 50 μm. The sections were directly transferred into Dounce homogenizers containing 0.5 mL of homogenization buffer and allowed to rest for 3 minutes to facilitate dissolution of the OCT compound. Following this, mechanical dissociation was performed in two steps: an initial coarse homogenization using a loose pestle, followed by fine homogenization using a tight pestle. The homogenized material was filtered through a 40-μm cell strainer to remove large debris. The resulting suspension was centrifuged at 500 × *g* for 6 minutes at 4°C, using an acceleration setting of 0 and deceleration setting of 3. The supernatant was carefully removed, and the pellet was resuspended in 0.5 mL of wash buffer. A nuclei count was then performed using a C-chip disposable hemocytometer. Samples were then subjected to a second centrifugation at 500 × *g* for 5 minutes at 4°C. After removal of this second supernatant, the pellet was resuspended in 100 μL of 10x Genomics 0.1× lysis buffer and incubated on ice for 2 minutes. Subsequently, 500 μL of 10x Genomics Wash Buffer 2 was added, and the mixture was gently mixed. A second nuclei count was performed using a C-chip disposable hemocytometer. The nuclei were pelleted again and resuspended to the target concentration for nuclei recovery (7,000 nuclei) in 10x Genomics Diluted Nuclei Buffer supplemented with 1 U/μL of protector enzyme. The preparation then proceeded according to the manufacturer’s instructions outlined in the Chromium Next GEM Multiome ATAC + Gene Expression User Guide (10x Genomics; Document CG000338).

### Alignment and processing of single-nuclei ATAC-seq data

Single-nuclei RNA-seq data were mapped to the GRCh38-2020-A human reference genome and quantified using the cellranger-arc pipeline (v2.0.1, RRID: SCR_023897). We also removed two patients with unusual clinical histories and samples with no cancer cells were removed for analyses. Technical replicates were merged per patient, and biological replicates (multiple pieces from the same tumor) were kept separate for analysis (sample ID as “-1” or “-2”). Only confidently labeled cells were included for analyses.

The gene expression component of the data was processed using scanpy (16) using the same processing steps as the single-cell data (above) but filtering genes expressed in fewer than 10 cells. Doublets called by Scrublet (17) were removed where they formed clear clusters, and cells were removed if they contained >50,000 UMIs. Cells were annotated based on the expression of canonical marker genes (Supplementary Fig. S1). Samples with no cancer cells were excluded from analyses.

The ATAC component of the data was processed separately after removing samples with a low % of fragments in cells or low % fragments in peaks (based on “error” thresholds provided by 10x). We processed raw fragments with SnapATAC2 (v2.8, RRID: SCR_026622; ref. 18) using the GRCh38 reference genome, in which basic quality control was performed per sample before combining all samples together for peak calling per cell label with MACS3 (RRID: SCR_013291, Cell labels: stroma, immune, osteosarcoma, desmoplastic small round cell tumour (DSCRT), fusion-negative RMS, fusion-positive RMS, and fusion-negative RMS cells from patients who died of their disease; ref. 19). Quality control included doublet removal, cell filtering (tsse >5), and feature selection to 50,000 features. Cell annotation labels were taken from the gene expression analyses in which cells were present in both the gene expression and ATAC components of the data. Any cells unique to the ATAC component (i.e., cells with only ATAC reads) were annotated by producing a gene matrix (ATAC reads that overlap genes) with the make_gene_matrix() function from SnapATAC2 that was used to cluster and look at canonical marker genes.

We calculated TF accessibility scores using pychromVAR (v0.0.4, RRID: SCR_027456) with TF motifs from the 2020 version of pyJASPAR (20, 21). Default settings of pychromVAR were applied to stromal, immune, converged (from fusion-positive and lethal fusion-negative patients), and diverse (from fusion-negative patients) cells. For plotting of ATAC coverage tracks, bigwig files were exported from SnapATAC2 and coverage and GTF tracks were visualized using pyGenomeTracks (19).

### DNA and RNA extractions for whole-genome and bulk RNA-seq

DNA was extracted from fresh-frozen tissue. Peripheral blood DNA was used as a matched normal, where possible. Total DNA and RNA were extracted using the AllPrep DNA/RNA Mini Kit (QIAGEN) following the manufacturer’s protocol. Frozen tissue samples, either as 50 μm sections of OCT-embedded material or approximately 3 to 20 mg of unembedded frozen tissue, were transferred into 2 mL Precellys homogenization tubes on dry ice. To each tube, 350 μL of Buffer RLT Plus supplemented with 2 mol/L dithiothreitol was added. Tissue homogenization was carried out using a Precellys 24 Touch Homogenizer at 6,000 rpm for 30 seconds. The homogenized samples were centrifuged at 10,000 rpm for 1 minute to remove any foam, and the supernatant was transferred to 2 mL Eppendorf tubes. Automated purification was then performed using the QIAcube (QIAGEN, RRID: SCR_020414), utilizing the kit-specific program for the AllPrep DNA/RNA Mini Kit. All steps were executed according to the default protocol preprogrammed into the QIAcube system to ensure optimal reagent handling, column processing, and elution conditions.

Upon completion of the extraction, two separate eluates were obtained per sample: one containing genomic DNA and the other total RNA. Each eluate was collected in 1.5 mL Eppendorf tubes. Quality control and integrity assessment were conducted using the Agilent 4200 TapeStation system (RRID: SCR_018435). Genomic DNA samples were evaluated using the Genomic DNA ScreenTape assay (Agilent, cat. #5067-5365), while RNA samples were assessed using the High Sensitivity RNA ScreenTape assay (Agilent, cat. #5067-5579) to determine RNA integrity number values. DNA samples were stored at −20°C, and RNA samples were stored at −80°C until further processing.

### Whole-genome sequencing and alignment

Genomic libraries with short inserts (∼500 bp) were prepared using the standard PCR-based whole-genome sequencing (WGS) library preparation protocol from Illumina. Flow cells were then loaded, and 150 bp paired-end sequencing clusters were generated on the Illumina NovaSeq 6000 platform. Sequencing was performed with a throughput of six samples per S4 flow cell lane. WGS output data were processed using standard tools from the Cancer Genomics Pipeline (CGP) to ensure high-quality and reproducible results. Sequencing reads were aligned to the GRCh38 reference genome using BWA-MEM v0.7.17 (RRID: SCR_010910), and duplicate reads were identified and marked using samtools markdup (RRID: SCR_002105). In all variant calling workflows below, for samples lacking a matched normal tissue, the *in silico* reference PDv38is_wgs was used.

### Variant detection and annotation

#### Point mutations and insertions and deletions

Somatic point mutations were identified using CaVEMan v1.15.2 (RRID: SCR_017089). Variants were filtered to retain only those with a PASS filter status and located in exonic or splice-site regions. Point mutations were additionally filtered by CLPM (median number of soft clipped bases in variant supporting reads) and ASMD (median alignment score of reads showing the variant allele) filters (CLPM = 0 and ASMD ≥ 140). VCF files were processed in R using the vcfR package. The resulting variants were matched against the COSMIC Mutant Census (22) v99 (GRCh38) using a composite key (chromosome, position, reference, and alternate alleles).

Small insertions and deletions (indel) were detected using Pindel (v01, RRID: SCR_000560). Annotated VCF files were filtered for variants with PASS status and parsed using the vcfR package (RRID: SCR_023453). Only indels located in protein-coding exons and predicted to cause nonsynonymous protein changes were retained. Variants were annotated with gene name, protein consequence, and classified by variant length and type. Cross-referencing with COSMIC (22) was performed to identify alterations in cancer genes (RRID: SCR_002260).

#### Structural variants

Structural variants (SV) were identified using BRASS v6.3.4 (CASM IT), GRIDSS (23) v2.13.1-gridss (RRID: SCR_027130), and GRIPSS v2.0 (HMFtools) to enhance sensitivity across variant classes. SVs from BRASS were processed using a custom R script, classified by variant type (deletion, inversion, tandem duplication, translocation, and amplification), and annotated with gene context using Ensembl gene coordinates (RRID: SCR_002344). Functional annotations were derived from COSMIC Cancer Gene Census (22), categorizing gene partners as fusion genes, oncogenes (OG), tumor-suppressor genes (TSG), or unclassified. A rule-based system prioritized SVs involving key cancer genes (e.g., deletions overlapping TSGs and tandem duplications affecting OGs).

In parallel, SVs were annotated from GRIPSS-filtered VCFs using the StructuralVariantAnnotation R package (RRID: SCR_018683). VCFs were imported and converted into GRanges and BEDPE formats, preserving fields like EVENTTYPE, AS, RP, and SR. Only autosomal or sex chromosomal SVs with PASS status were retained. Intrachromosomal SVs were filtered for size (>500 bp) and allele scores (≥300 for 500–30,000 bp; ≥250 for >30,000 bp); interchromosomal events required allele scores ≥300. All SVs needed ≥4 supporting read pairs and ≥2 split reads. SV breakpoints were overlapped with Ensembl gene coordinates and assessed for involvement with COSMIC genes (22). High-confidence SVs were defined by thresholds of ≥50 RP or ≥25 SR, or both for stringent selection.

#### Copy-number alterations

Copy-number analysis was conducted using tools from the Hartwig Medical Foundation toolkit. AMBER (v4.1.1, RRID: SCR_027131) and COBALT (v2.0) were used to estimate allele-specific copy number and coverage, respectively. Final segmentation and interpretation were performed using PURPLE (v4.1, RRID: SCR_022999), integrating B-allele frequency and read depth.

Somatic copy-number segments were functionally annotated using COSMIC Cancer Gene Census genes (22). Segments were classified into gains (copy number >2.5), losses (<1.5), copy-neutral loss of heterozygosity (1.7–2.4 total copy number with <0.5 minor allele copy number), and complex gain–loss regions. Recurrently altered regions were identified based on frequency across samples and summarized at the gene and cytogenetic band levels.

Adjacent segments with identical copy-number status and contiguous genomic coordinates were merged to define larger alteration blocks. These were cross-referenced with COSMIC annotations (22) to assess whether the observed changes occurred within broader copy-number variation regions or represented isolated breakpoint-level events. The size and classification of each event were recorded accordingly.

### RNA processing for bulk RNA-seq

Paired-end FASTQ files corresponding to each sample were identified and processed. Reads were quality and adapter trimmed using BBDuk (RRID: SCR_016969). Trimmed reads were aligned to the human reference genome using STAR (RRID: SCR_004463; ref. 24) with ENCODE-recommended RNA-seq alignment parameters. The STAR genome index used was based on the GRCh38_v32 annotation (RRID: SCR_014966). Strand-specificity was assessed using STAR-generated ReadsPerGene.out.tab files. Total read counts mapping to forward and reverse strands, as well as unstranded reads, were calculated. Strand bias ratios were used to classify libraries as unstranded, forward-stranded, or reverse-stranded.

Expression levels (raw counts) were estimated using three methods:

1. RSEM (RRID: SCR_000262): Transcript- and gene-level expression quantification was performed using the STAR-generated transcriptome BAM file. Strand-specific parameters were passed based on the strand classification results.
2. featureCounts (RRID: SCR_012919): Uniquely and concordantly mapped read pairs were extracted from the coordinate-sorted BAM using samtools and then name-sorted. Gene-level counts were computed using featureCounts with the GTF annotation file, and strand information was appropriately passed.
3. Salmon (RRID: SCR_017036): Two Salmon-based quantification strategies were used. First, selective alignment to the transcriptome was performed directly from the trimmed FASTQ files using Salmon –validateMappings mode. Second, quantification was conducted from the transcriptome-aligned BAM file using the –useAlignment mode.

Putative gene fusions were identified using STAR-Fusion (RRID: SCR_025853). Trimmed paired end reads previously processed using BBDuk were used as input. STAR-Fusion was run with default parameters and configured to validate fusions using FusionInspector, reconstruct candidate fusion transcripts *de novo*, and assess the potential coding impact of each fusion event.

Processing of raw counts was performed using edgeR (v3.42.4, RRID: SCR_012802; ref. 25), which includes library-size normalization and log-transformation to provide logCPM values. Genes were retained only if they had a logCPM >0.5 in at least 30% of cases. Gene module scoring of all bulk RNA samples was performed using singscore (v1.20; ref. 26).

### Processing of external datasets

Raw count data from three projects within the Human Cell Atlas (RRID: SCR_016530; refs. 27–29) were combined and processed together using scanpy (as above; ref. 16) after removing genes expressed in fewer than 15 cells. This combined fetal limb atlas was used to derive signatures for mesenchymal lineages.

We downloaded raw single-cell or single-nuclei RNA-seq counts for three cohorts: Massachusetts General Hospital (11), St Jude (10), and Princess Maxima Center (13). Datasets were processed separately using scanpy (v1.11.3), including filtering genes that were expressed in fewer than 10 cells, CPM normalization, and log-transformation. We restricted our analysis to primary RMS tumor samples, excluding cell lines and xenografts (patient-derived xenografts), to best reflect our cohorts and to confirm presence of the converged cell state *in vivo*.

We obtained raw bulk RNA-seq counts for three cohorts: NIH (6), St Jude (30), and Princess Máxima Centre (31). For each cohort, we processed raw counts using edgeR (v3.42.4, RRID: SCR_012802; ref. 25), including filtering of lowly expressed genes and logCPM calculation. All scoring of modules in bulk RNA samples was performed using singscore (v1.20; ref. 26). All samples were derived from primary tumor samples with the exception of the patient-derived tumoroid bulk data from Meister and colleagues (31), which were found to recapitulate fusion-positive and -negative phenotypes.

### Derivation of mesenchymal lineage gene modules using the human cell atlas

To obtain specific gene modules for each mesenchymal lineage from the fetal atlas (27), we first split the “osteochondral” cell label into “cartilage lining” [interzone 1 and 2 cells, articular chondroprogenitor cells (CC)], “cartilage hypertrophic” (resting CCs, hypertrophic CCs, prehypertrophic CCs, proliferating CCs, early resting CCs, cycling CCs, and cycling progenitor cells), “bone cortical” (mature periosteum, preosteoblast 1 and 2, perichondrium, early periosteum, MMP9 periosteum, and osteocyte), and “bone osteoblast” (mature osteoblast, early osteoblast, and HEY1 osteoblast) as we expected unique expression signals from these categories (Supplementary Table S2). We used the Wilcoxon method in scanpy’s rank_genes_groups function to identify genes specific to each cell type. We extracted the top 500 genes separately for either sequencing batch (5′ or 3′) and took the intersection per cell type category. Using the top genes per category, we further filtered to genes that were expressed in a large percentage of cells in each cell type category and a low percentage in other unrelated cell types. To ensure genes were unique to each cell type, any overlapping genes between modules were removed. Pseudogenes and long noncoding genes were also removed.

To calculate the mesenchymal “other” composite score, we took the average z-standardized score of five modules—tendon, fibrous, cartilage (lining), cartilage (hypertropic), and bone (cortical)—excluding modules that were either biologically irrelevant or had low expression across patients. Scores were z-standardized alongside reference and other tumor cells (Ewing sarcoma, DSCRT, and osteosarcoma) to contextualize them. The convergence metric was then the log_2_ ratio between the muscle score and the composite, in which cells were considered “converged” if they had a convergence metric >0. As the muscle module contains only 10 genes, the convergence metric offers a robust way to quantify the convergence onto muscle, relative to other mesenchymal signals, so that it could be applied across data modalities and cohorts. The convergence metric relies on more genes (improving score stability), adjusts for heterogeneous mesenchymal backgrounds, and normalizes muscle identity relative to individual mesenchymal baselines to reduce technical variance across modalities.

### Derivation of high-risk gene module using our discovery cohort

To derive a high-risk gene module, we looked for genes that were upregulated in the high-risk patient cluster relative to fusion-negative patients with a good outcome. We were prevented from performing a pseudobulk differential expression analysis as normalization could not account for the vastly different cell numbers in our relatively small cohort. We instead opted for a differential proportions analysis to assess which genes increase in the proportion of cells expressing them between fusion-positive or lethal fusion-negative patients and fusion-negative patients. We removed samples with fewer than 200 cells (removing the fusion-negative alveolar patient) and filtered genes based on a mean proportion ratio of ≥2 to prioritize genes with larger absolute differences. We ensured specificity of expression by filtering to genes expressed in >15% of cells in at least half of high-risk patients and <15% of cells in at least half of non–high-risk patients. We also removed long noncoding genes, ribosomal genes, and lowly expressed genes. Genes are annotated based on whether they are associated with a muscle lineage, neural lineage, or a mixed/other lineage. For a gene to be annotated as either muscle or neural, they needed to demonstrate lineage specificity in the developing spinal atlas, expression in the Human Protein Atlas (HPA; ref. 32), and have literature support. Overrepresentation analysis with “Biological Process” terms from Gene Ontology (GO; RRID: SCR_002811) was performed using default settings (minGSSIze = 10, maxGSSize = 500) in clusterProfiler (33). *P* values were corrected using Benjamini–Hochberg correction.

### Differential expression analysis of the extension cohort

We used the extension cohort to perform a pseudobulk differential expression analysis between converged and diverse cells. Cells were considered converged if they had a convergence metric >0 and were either from a fusion-positive patient or a fusion-negative patient who died of disease. Cells were considered diverse if they had a convergence metric <0 and were from a fusion-negative patient who did not die of their disease. Patients with fewer than 250 cells were removed. Counts were aggregated into pseudobulk profiles per sample providing 15 “converged” profiles from patients who died of disease and 12 “diverse” profiles from patients who did not. We summed across replicate samples using the *sumTechReps()* function. Genes were retained if they had a logCPM of ≥0.5 in at least 20% of samples. Using a quasi-likelihood framework in edgeR (v3.42.4; ref. 25), we identified differentially expressed genes based on an FDR threshold of 0.05 with Benjamini–Hochberg *P* value correction. Of the 1,826 genes upregulated in converged cells, 30 were TFs that were present in the JASPAR database used by chromVAR (20, 21). TFs were annotated as either muscle or neural based on lineage specificity in the developing spinal atlas and the HPA (32).

### PAX3–FOXO1 overexpression in fusion-negative RMS tumoroid models

The PAX3–FOXO1 fusion or tdTomato cDNA were cloned into pLV-EF1a-[cDNA]-PGK-puro (obtained from VectorBuilder: pLV-EF1a-PAX3FOXO1-PGK-puro (Vector ID: VB220603-1030fex) and pLV-EF1a-tdTomato-PGK-puro (Vector ID: VB900122-1868kvz). Lentiviruses were produced in HEK293T cells (RRID: CVCL_0063) using standard packaging protocols.

Fusion-negative RMS models RMS000OYW (Tumoroid 1) and RMS444 (Tumoroid 2) were previously established and cultured as described (31). For lentiviral transductions, fusion-negative RMS cells were seeded in six-well plates and infected with either pLV-EF1a-PAX3FOXO1-PGK-puro or pLV-EF1a-tdTomato-PGK-puro lentiviruses in the presence of polybrene (34). Following antibiotic selection, cells were cultured for 17 to 24 days after selection, harvested, and viably cryopreserved for downstream single-cell analysis.

### Derivation of the fusion gene module from tumoroid fusion experiments

For the derivation of the fusion module, we were able to conduct a pseudobulk differential expression analysis owing to the larger number of cells and clarity of signal in the tumoroid single-cell data. First, we only considered a cell fusion-positive if it was induced for the fusion and had nonzero expression for at least two downstream targets of PAX3:FOXO1 (KCNN3, MCAM, MYOD1, GREM1, FGFR4, and MET; ref. 35). We considered cells in the control samples to be fusion-negative if they had zero expression for all six targets above. We ensured doublet removal by only keeping cells with a total count of less than 50,000. For samples with more than 2,000 cells, we performed k-means clustering (k = 3) to obtain smaller pseudobulk samples.

We aggregated all cells across each sample to obtain pseudobulk profiles and performed differential expression analysis using edgeR (v3.42.4; ref. 25). We removed mitochondrial genes and retained genes with a logCPM ≥0.5 in at least 10% of samples. A quasi-likelihood framework was applied to identify differentially expressed genes between control and fusion-positive cells while accounting for the effect of the tumoroid. We determined significant genes using Benjamini–Hochberg *P* value correction and an FDR threshold of 0.05.

### Sample processing for *in situ* gene expression profiling with Xenium

Fresh-frozen tissue samples were embedded in OCT (Tissue-Tek OCT. Compound, 25608-930) and cryosectioned on a Leica CM1950 cryostat. Ten-micrometer sections were mounted onto a Xenium Slide (10x Genomics, PN 3000941) and stored at −80°C according to the 10x Genomics sectioning protocol (CG000579). Prior to probe hybridization, tissue sections were thawed, formaldehyde-fixed, and permeabilized with 70% methanol (10x Genomics protocol CG000581) to allow probe penetration while preserving tissue morphology.

The Xenium 5K Gene Expression Panel (PN-1000724) was applied to the tissue sections and incubated overnight (between 16–24 hours) using an Analytik Jena Biometra TAdvanced 96 SG thermal cycler with 96-well block with a Xenium Prime adapter. The hybridization step was performed according to the 10x Genomics Xenium protocol (CG000760) and using 10x genomic Xenium Prime sample preparation kit (PN-1000720), allowing specific padlock probe binding to RNA targets. Following hybridization, the signal was ligated and amplification steps were conducted via rolling circle amplification to generate tagged products for imaging on the Xenium Analyzer. After amplification is complete, the sections were treated with Xenium Cell Segmentation Staining Reagents (PN-1000661) overnight incubation at 4°C (16–24 hours). 4′,6-diamidino-2-phenylindole (DAPI) staining was included to visualize nuclei and assist in cell segmentation.

Amplified signals were detected using high-resolution fluorescence imaging with the Xenium Analyzer system (RRID: SCR_023910). Multiple rounds of imaging were performed to decode the barcoded probes, capturing spatially resolved transcriptomic data across the tissue sections. Once the run completed, samples were placed into fresh PBS-T until post-Xenium staining occurred [hematoxylin and eosin (H&E)].

### Xenium quality control and data processing

Xenium spatial transcriptomics data were processed and quality controlled using a Squidpy (36) and a custom Python workflow. Data were filtered on a per-sample basis using sample-level quality control thresholds. The median and median absolute deviations (MAD) were calculated for both “total_counts” and “*n*_genes_by_counts” per sample. A lower threshold for “total counts” was set to either 10 or three MADs below the sample-specific median, whichever was greater. A separate threshold for detected genes was calculated similarly as three MADs below the sample-specific median. Subsequently, genes expressed in fewer than five cells were removed from each sample. Cells were further filtered to retain only those with a number of detected genes greater than the calculated threshold and those annotated with exactly one nucleus. After filtering, all samples were concatenated into a unified AnnData object using the sample identity as the batch key, with batch categories set to the sample names. The resulting filtered dataset was used for downstream analysis.

Dimensionality reduction was performed using PCA and UMAP, followed by Leiden clustering using Scanpy. Gene signature scoring was applied to identify myeloid, vascular, extracellular matrix–related, and lymphoid populations using curated gene sets. Putative cancer clusters were identified based on Leiden cluster identities and downstream interpretation of gene expression patterns.

To define a Xenium cancer cell, cells must express either canonical myogenic marker, *MYOD1* or *RYR1*. Gene modules related to the high-risk signature was used to score cells using Scanpy’s score_genes function. UMAP embeddings were visualized and annotated by sample and custom-defined outcome groups. Boxplots of outcome and high-risk scores were generated across sample groups using Seaborn (RRID: SCR_018132).

### Spatial neighborhood enrichment and calculation of autocorrelation metrics

Spatial neighborhood analyses were performed using the Squidpy package (36). Spatial neighbor graphs were constructed using a Delaunay triangulation approach. Centrality scores were calculated based on Leiden clusters. To reduce computational load, the dataset was subsampled to 80% of cells. Neighborhood enrichment analysis was then performed on the subsampled data to assess intercluster spatial co-occurrence. To assess spatial gene expression patterns, Moran I and Geary C statistics were computed using 100 permutations each. Genes exhibiting significant spatial autocorrelation (Moran I >0.1 and FDR-adjusted *P* < 0.05) were identified.

### Identification of cell-surface targets

We calculated the proportion of cells expressing each gene within predefined groups, defining expression as any value greater than zero. For each group, the percentage of cells expressing each gene was computed by dividing the number of expressing cells by the total number of cells in that group. Genes expressed in fewer than 10% of stromal and immune cells from our RMS atlas were identified to exclude those broadly expressed in normal, nonmalignant populations. To enrich genes’ characteristic of aggressive disease, genes expressed in more than 10% of cells from both fusion-positive high-risk (fusion-positive HR) and fusion-negative high-risk (fusion-negative HR) groups were selected. Genes expressed in more than 25% of cells from either high-risk genotype were also considered. These thresholds were intended to identify genes consistently active across high-risk RMS subtypes.

Differentially expressed genes upregulated in high-risk RMS (e.g., fusion-positive and lethal fusion-negative) were selected based on test statistics derived from differential expression analysis. Genes with a test score greater than 50 and a positive log fold change (FC) were retained, indicating strong statistical support and higher expression in the query group. We compared a specified “query” group against either a “reference” group or all other groups combined. Cells were assigned to either the query or reference group accordingly. Statistical testing for differential expression was conducted using the Wilcoxon rank-sum test implemented in Scanpy’s rank_genes_groups function. The analysis generated statistics including gene ranking scores, log FCs, and both raw and adjusted *P* values. Genes were then ranked primarily by adjusted *P* value and log FC to identify the most significant differentially expressed genes.

Oncofetal targets, genes, or proteins normally expressed during fetal development but largely silenced in healthy adult tissues and reexpressed in cancer, were identified by assessing candidate genes within the fetal muscle data from the Human Cell Atlas (27). Genes expressed in more than 5% of fetal muscle cells from a fetal tissue atlas were identified. Candidates were then cross-referenced against the Genotype-Tissue Expression (GTEx) database (37). Gene-level read counts were obtained from the GTEx v10 RNA-seq dataset and processed using the edgeR package (25). Normalization factors were computed using the trimmed mean of M values method. LogCPM were calculated for all genes and averaged across tissues. Expression levels were categorized into five bins based on mean logCPM values: extremely low (≤0), very low (>0 and ≤1), low (>1 and ≤3), moderate (>3 and ≤4), and high (>4). For each gene, the number and identity of tissues falling into each expression category were recorded. Additionally, the proportion of tissues with logCPM >1 was computed per gene to assist in prioritization.

Cell-surface proteins were prioritized due to their accessibility to CAR T cells or antibody-based therapies, which could facilitate rapid clinical translation. Cell-surface protein candidates were identified by integrating multiple curated databases. Subcellular localization data from the HPA was first processed, and genes annotated with membrane-related terms in the “main location” field were selected. The GO database was parsed using the goatools library (RRID: SCR_025305) to extract term names and evidence codes for GO terms. Genes annotated with the GO terms cell surface (GO:0009986) and plasma membrane (GO:0005886) were retrieved from associated files. Only GO annotations supported by strong experimental evidence (e.g., EXP, IDA, IPI, IMP, IGI, and IEP) were retained, and UniProt identifiers (RRID: SCR_002380) were mapped to gene names using a comprehensive UniProt ID mapping file. Additionally, receptor proteins were extracted from the CellPhoneDB resource (38). Proteins annotated as receptors were selected, and the corresponding gene names were obtained. A final list of candidate cell-surface genes was compiled by combining genes from the HPA membrane-associated dataset, GO-based annotations, and CellPhoneDB receptor proteins.

Known drug–target associations were obtained from the Open Targets platform (RRID: SCR_02170) by reading two Parquet files corresponding to different partitions of the “known_drug” dataset. For each matched gene, all associated drug types and drug names were extracted and stored. A “hit” in Open Targets was considered as supporting evidence linking the gene or protein to disease pathways, thereby strengthening its candidacy for therapeutic development. Certain drugs from Open Targets were identified as unsuitable for repurposing; however, they serve to illustrate the druggability of their respective targets.

### Proteomic validation of cell-surface targets

Formalin-fixed, paraffin-embedded tissue blocks from 10 RMS cases were sectioned at 3 μm and subjected to IHC staining on the Leica Bond platform (RRID: SCR_025548). H&E and myogenin stains were performed as reference assays. Antibodies were selected pragmatically on the basis of availability and prior validation and included FGFR4 (Santa Cruz Biotechnology, SC-136988, clone A-10, monoclonal, lot J1123, 1:75) and CDH15 (Invitrogen, PA5-11489, polyclonal, lot 80052356, 1:75, RRID: AB_2260299). Staining was performed using the Leica Bond IHC F protocol with 3,3′-diaminobenzidine (DAB) enhancer, with an additional protein block included for CDH15. Staining was reviewed qualitatively for extent and pattern of tumor-cell positivity.

### Assessment of fetal acetylcholine receptor expression by flow cytometry

RMS cell lines RH30 (RRID: CVCL_0041) and RH41 (RRID: CVCL_2176) were obtained from DSMZ, whereas RD (RRID: CVCL_1649), TE381.T (RRID: CVCL_1751), and TE441.T (RRID: CVCL_1754) were obtained from the ATCC. No authentication beyond that provided in the supplier certificate of analysis was performed. At the time of purchase, a master cell bank was established for each line, all banks tested negative for *Mycoplasma*, and aliquots from these master banks were used for the experiments presented here. Dissociated RMS tumor tissue was collected during sample processing for scRNA-seq and, where sufficient material was available, was also used for flow cytometric analysis. For flow cytometry, cells were stained for B7-H3 using the MIH42 antibody (BioLegend, RRID: AB_10717510). Detection of the fetal acetylcholine receptor isoform containing the γ subunit encoded by *CHRNG* was performed using mAb131 (39), generated by transfection of 293T cells with plasmids encoding the heavy- and light-chain variable regions of the antibody. As indicated, mAb131 was used either directly as culture supernatant or after concentration with Amicon Ultra-15 centrifugal filter units (100 kDa molecular weight cutoff; Merck Millipore), followed by purification using the rProtein A GraviTrap system (GE Healthcare) and Antibody Buffer Kit (Cytiva). Antibody-stained cells were analyzed on an LSR II flow cytometer (RRID: SCR_002159) using FACSDiva software v6.0 (RRID: SCR_001456), and data were processed in FlowJo v10.8 (BD Biosciences, RRID: SCR_008520).

## Results

### Overview of the discovery and extension cohorts

**Figure 1.**
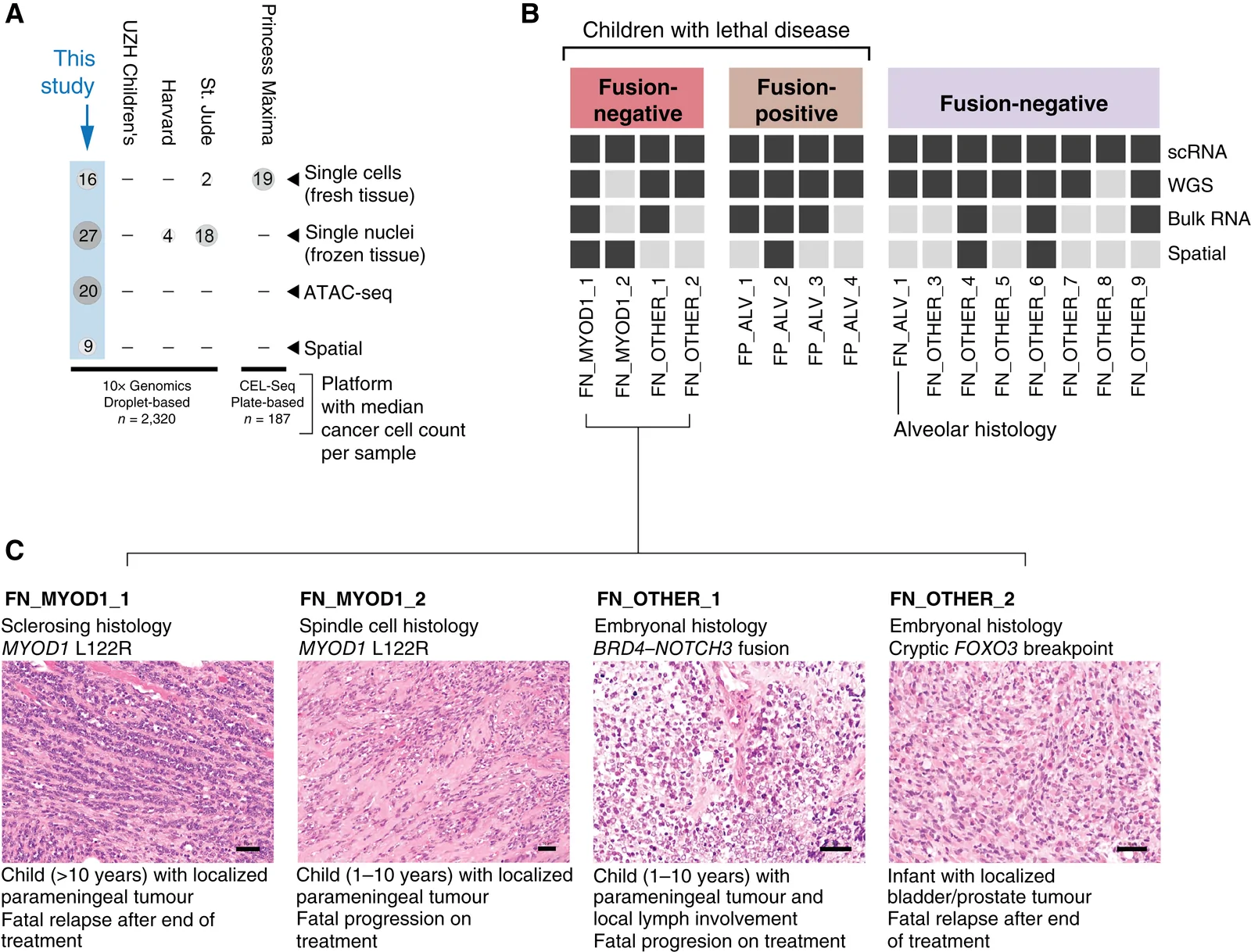
Multiomics profiling and clinical characterization of RMS across discovery and extension cohorts. **A,** A summary of previously published single-cell analyses of primary RMS. The current study (highlighted in blue) uniquely performed high-throughput single-cell transcriptomic profiling on fresh tissue, which preserves native gene expression states while minimizing artifacts caused by freezing. Sample numbers for our cohort only include samples that contain RMS cells. **B,** Heatmap depicts the application of sequencing assays across individual patients in the discovery cohort. Patients are grouped by clinical category: fusion-positive alveolar RMS (brown), fusion-negative with fatal outcome (red), and fusion-negative that have survived (purple). Each column represents an individual patient. The data indicates whether single-cell RNA-seq, WGS, and RNA-seq were performed. **C,** H&E-stained tumor sections from patients with fusion-negative RMS who died of their disease. These cases highlight histologic diversity and aggressive clinical behavior despite the absence of characteristic gene fusions.

We assembled a discovery cohort of 16 children with RMS, representative of the disease spectrum in terms of fusion status, histologic subtypes, stage, and anatomic site of the primary tumor (Supplementary Table S1). The median follow-up time of surviving children in this cohort was 44 months. Previous studies in the field often relied on either frozen tissue or lower-throughput single-cell approaches. In contrast, we obtained fresh tissue from each tumor and generated high-throughput single-cell mRNA data (Fig. 1A). For comparison, we included two children with Ewing sarcoma in the discovery cohort. For validation, we assembled an extension cohort of 27 historic archival samples (fresh-frozen tissue) from 24 children, which we studied by multiomics single-nucleus RNA and ATAC sequencing. In this study, we included one desmoplastic small round cell tumor (DSRCT) and two osteosarcomas for comparison, normalization, and to provide baseline sarcoma expression in which a muscle signal is not expected. In addition, we obtained high-resolution spatial mRNA readouts from nine samples processed by Xenium (single-cell resolution). Where feasible, we characterized tumors using WGS and bulk RNA-seq. We provide an overview of primary RMS samples and assays used in this study in Fig. 1B with further details in Supplementary Table S1.

### Children with fusion-negative lethal tumors

**Figure 2.**
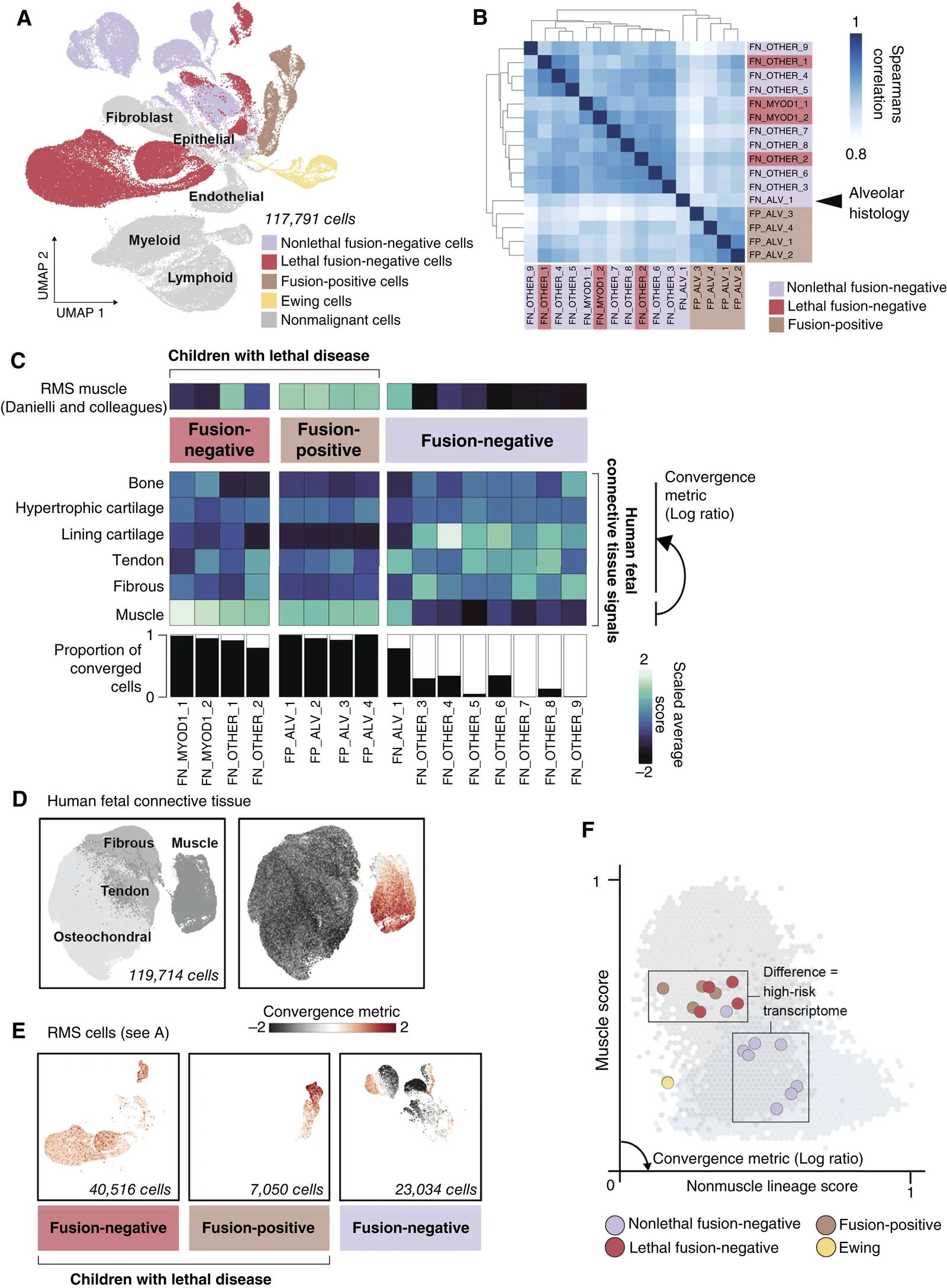
Fusion-positive and lethal fusion-negative cells converge on a high-risk transcriptome. **A,** UMAP representation of 117,791 cells across all patients in the discovery cohort. Dots are colored by malignant cell type, and normal cell type clusters are indicated by labels. Includes cells from four fusion-positive patients (brown), 12 fusion-negative patients (red and purple), and two patients with Ewing sarcoma (yellow). **B,** Heatmap showing the hierarchical clustering of aggregated patient profiles of cancer cells across the entire transcriptome. Hierarchical clustering is based on pairwise Spearman correlation. **C,** Heatmap showing the average scores per patient for various gene modules, including the “muscle lineage” module derived by Danielli and colleagues (top) and for each of the mesenchymal modules (middle). Below the heatmap we show the proportion of converged cells per patient (bottom). Converged cells are defined as having a convergence metric greater than zero (see Materials and Methods). Scores have been scaled and mesenchymal modules are detailed in Supplementary Table S2. Text annotations describe that the convergence metric is calculated as the log ratio of muscle score over nonmuscle mesenchymal score per cell. **D,** UMAP representation of 119,714 cells from the fetal connective tissue atlas, only cell types represented in the convergence metric (bone cortical, cartilage hypertropic, cartilage lining, tendon, and fibrous) are included in the UMAP. Dots are colored by convergence metric to contextualize the range of metric scores. **E,** UMAP from (**A**) but colored by convergence metric in separate facets for each malignant cell group: RMS cells from fusion-negative lethal cases, from fusion-positive cases, or from fusion-negative cases. **F,** Score landscape showing muscle lineage score against a composite nonmuscle lineage score (cartilage lining, fibrous, tendon, cortical bone, or cartilage hypertrophic), averaged per patient and colored by if the cancer cells come from a patient with fusion-positive RMS (brown), lethal fusion-negative RMS (red), fusion-negative RMS (purple), or Ewing sarcoma (yellow). Honeycomb background represents scores for either muscle (gray) or nonmuscle mesenchymal lineages (blue) from the fetal atlas used to derive the modules. All scores have been scaled. Text annotations describe that the convergence metric is calculated as the log ratio of muscle score over nonmuscle mesenchymal score per cell, and the high-risk transcriptome is derived from a comparison between the “high-risk” cluster (fusion-positive and lethal fusion-negative RMS cells, excluding the fusion-negative alveolar case) and fusion-negative RMS cells (see Materials and Methods).

The discovery cohort comprised four children with lethal, fusion-positive and 12 with fusion-negative RMS. Amongst these 12 children, four children with non-alveolar localized tumors who died of disease were included (see Supplementary Table S1 for genetic features): two children with tumors harboring a canonical *MYOD1* mutation, a variant that has been associated with unfavorable outcomes; a child with an embryonal tumor carrying a rearrangement breakpoint within the *FOXO3* gene (not *FOXO1*) with no associated fusion partner; and a child with localized RMS harboring an in-frame *BRD4–NOTCH3* gene fusion, a rare variant that has been described in a few RMS tumors (40). All four children were not considered to have clinically actionable molecular high-risk features (Fig. 1C), as per European practice. Based on fusion status and outcomes, we subdivided the discovery cohort into three groups: children with fusion-negative lethal disease (*n* = 4), children with fusion-positive disease (*n* = 4), and children with fusion-negative disease who remain alive (*n* = 8). We processed fresh tissue samples of this discovery cohort within 24 hours of surgical resection to generate single-cell transcriptomic data using standard procedures (see “Materials and Methods”). We annotated cells based on canonical markers (Supplementary Fig. S1A) and evidence of cancer-specific copy-number changes (Supplementary Fig. S1B and S1C; ref. 41). Accordingly, we identified 70,600 RMS cancer cells (median of 2,602 per case) and 44,098 noncancer cells (median of 683 cells per case; Fig. 2A).

### Cells from high-risk tumors converge on a common transcriptome

First, we performed conventional hierarchical clustering of cancer cells. This analysis reproduced the well-established segregation of RMS by fusion status (6): fusion-positive tumors formed one clade, whereas fusion-negative RMS, including lethal tumors, formed the other (Fig. 2B). A rare case of fusion-negative, alveolar RMS formed a separate subcluster within the clade of fusion-positive tumors (which are exclusively alveolar). Similarly, when we scored muscle differentiation using a recently published muscle gene signature that was derived from RMS single-cell transcriptomes (14), tumors segregated largely by fusion status but not by disease outcome (Fig. 2C). However, on assessment of differentiation signals derived from human fetal connective tissues (27–29), lethal fusion-negative tumors instead converged with fusion-positive cancers. Given that RMS cells derive from connective tissue, we assessed the degree to which cancer cells exhibited signals of human fetal muscle, tendon, fibrous tissue, lining cartilage, hypertrophic cartilage, or cortical bone by identifying gene lists (“modules”) that define each lineage in a single-cell transcriptomic map of human fetal connective tissues (*n* = 249,151 cells; Supplementary Table S2). We found, as expected, that fusion-positive tumors exhibited strong muscle differentiation and decreased signals of other mesenchymal lineages (Fig. 2C). Fusion-negative tumors of children who are alive, on the other hand, resembled mesenchymal lineages other than muscle more closely, except for the fusion-negative tumor with alveolar histology. Lethal fusion-negative tumors, like fusion-positive cases, displayed a strong muscle signal.

Next, we quantified the relation of muscle versus non-muscle mesenchymal differentiation of each tumor by scoring individual cells by their differentiation (log ratio of muscle vs. aggregated other differentiation signals), which we refer to as the “convergence metric” (Fig. 2D and E). This metric reflects the convergence of lethal-outcome tumors, despite distinct underlying genetic alterations, on a muscle-like transcriptome with reduced connective tissue signals. We found that at the resolution of single cells, lethal, fusion-negative tumors again resembled fusion-positive tumors. A high proportion of cells exhibited an increased convergence metric, a state we termed “converged” [Fig. 2C (bottom); *P* = 3.11 × 10^−4^; two-sided Mann–Whitney U test]. By contrast, in fusion-negative tumors with non-lethal outcomes, cells exhibited a diverse range of differentiation profiles (“diverse” state). Therefore, lethal fusion-negative, non-alveolar tumors did not resemble embryonal tumors with good outcomes, but instead, they bore a striking similarity to fusion-positive tumors.

Lastly, we projected our tumor samples onto a two-dimensional map of fetal normal cells (represented by the honeycomb background) based on their differentiation signals, allowing us to visualize tumor similarity to fetal cell types within a defined mesenchymal space (Fig. 2F). This analysis segregated tumors by outcome, fusion status, and histology: one cluster comprised fusion-negative tumors with favorable outcomes. In the other cluster lethal, fusion-negative tumors and fusion-positive tumors (along with the one alveolar, fusion-negative case) congregated. By contrast, Ewing sarcoma cells were absent for expression signals in this analysis, consistent with previous reports of a lack of lineage-specific mesenchymal differentiation in Ewing sarcoma (42). The transcriptional difference between tumor clusters, fusion-negative versus cells from fusion-positive and lethal fusion-negative tumors (see Materials and Methods), defined a “high-risk” transcriptome of genes upregulated in converged cells (*n* = 89 genes; Supplementary Table S3). We found this score to be associated with higher risk categories even when defined by older, non–fusion-based risk assessment (Supplementary Fig. S2; ref. 43). Collectively, these findings show that lethal fusion-negative tumors more closely resemble *FOXO1* fusion-positive RMS, suggesting that an overarching high-risk transcriptome may exist.

### Validation in an extension cohort and in external data

**Figure 3.**
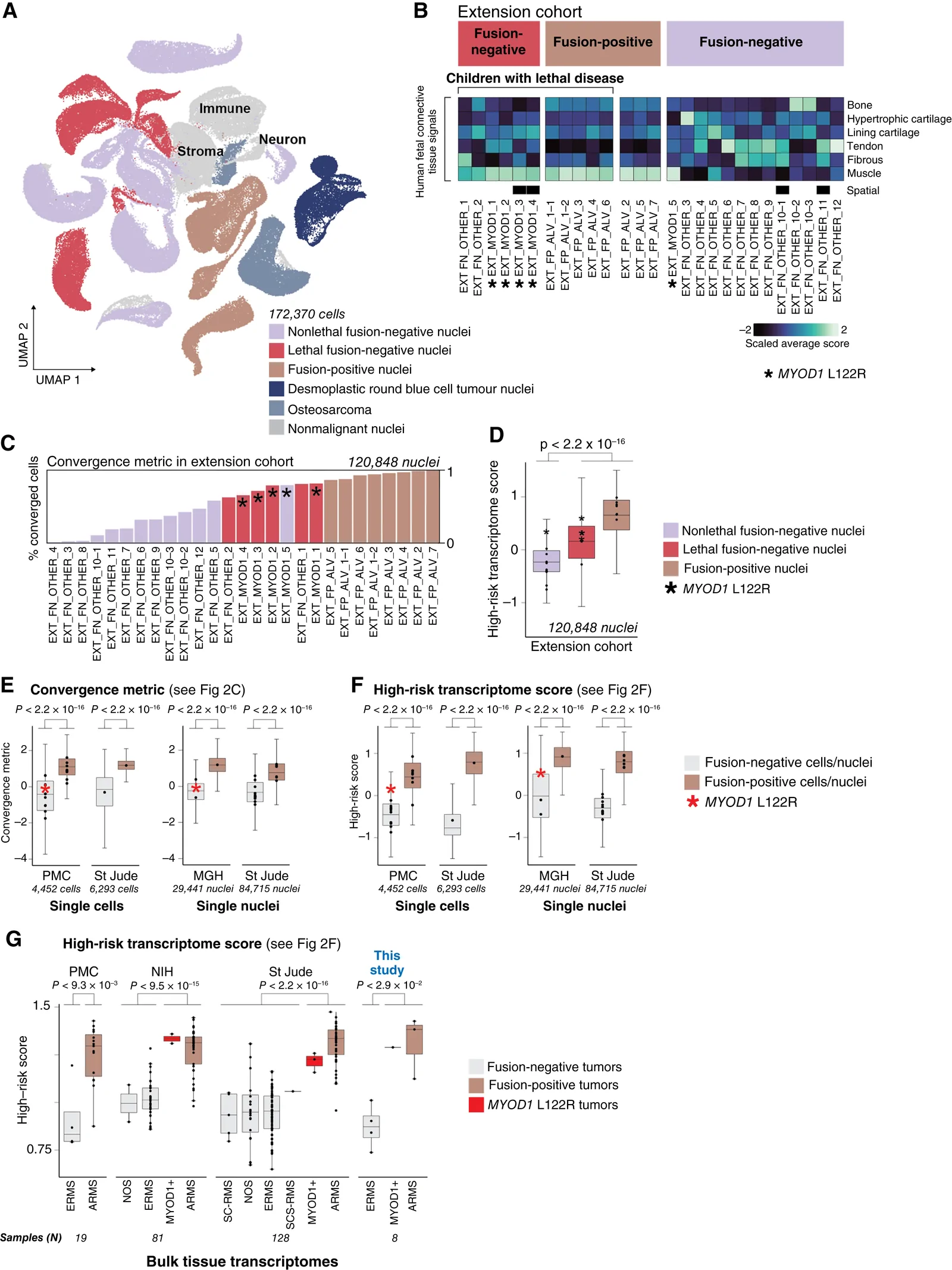
Evidence of converged cells in fusion-positive and lethal fusion-negative patients across modalities and cohorts. **A,** UMAP representation of 172,370 nuclei across all patients in the extension cohort. Dots are colored by malignant cell type, and normal cell type clusters are indicated by labels. Includes nuclei from seven fusion-positive patients (brown), 17 fusion-negative (red and purple), two osteosarcomas (light blue), one desmoplastic small round cell tumor (dark blue), and one isolated myofibroma (no malignant nuclei). **B,** Overview of the “extension” cohort, with patients with RMS grouped according to whether they are fusion-negative but died of their disease (red), fusion-positive (brown), or fusion-negative (purple). The heatmap shows the average scores in RMS nuclei per patient for each of the mesenchymal modules. The row beneath the heatmap indicates whether spatial transcriptomics was performed, and patients that harbor *MYOD1*-mutant cells are indicated by an asterisk. Scores have been scaled and mesenchymal modules are detailed in Supplementary Table S2. **C,** Boxplots showing per-nuclei scores for the high-risk module across RMS cells from fusion-positive cases (brown), lethal fusion-negative cases (red), and nonlethal fusion-negative cases (purple). The dots represent the average score per patient, and the asterisks indicate that the patient’s cells are *MYOD1*-mutant. Patients with fewer than 50 RMS nuclei were removed. **D,** Bars indicate the proportion of cells per patient that are transcriptionally converged, in which a converged cell is defined as a cell with a convergence metric greater than zero. Bars are colored by whether the patient is fusion-positive (brown), fusion-negative (purple), or fusion-negative and died of their disease (red). Patients who harbor *MYOD1*-mutant cells are indicated by an asterisk. **E** and **F,** Boxplots showing the per-cell scores for either the convergence metric (**E**) or the high-risk module (**F**) across two external single-cell (10, 13) and two single-nuclei (10, 11) datasets. Boxplots are colored by whether the sample was from a fusion-negative (light gray) or fusion-positive (brown) patient. The dots represent the average score per patient, and the red asterisks indicate that the patient’s cells are *MYOD1*-mutant. **G,** Boxplots showing per-sample scores for the high-risk module across bulk RNA samples from three external datasets (6, 30, 31) and our cohort (“this study”). Boxplots are colored by whether the sample was taken from a fusion-negative patient (light gray), fusion-positive patient (brown), or a patient harboring a *MYOD1* mutation (red). Each patient is represented by a dot and *P* values were calculated using a two-sided Wilcoxon test.

To validate and extend this finding, we obtained 27 fresh-frozen tissues of 24 children from a national tissue bank (VIVO Biobank) and institutional archives and applied single-nucleus RNA and ATAC sequencing (Fig. 3A). An overview of the extension cohort by fusion status, outcome, *MYOD1* mutation status, and alveolar histology is shown in Fig. 3B. Following standard data processing (see “Materials and Methods”), we obtained 172,370 nuclei, of which 120,849 nuclei (median of 2,483 per case) represented cancer cells, as assessed from canonical markers and evidence of somatic genetic changes in transcriptomic data. We present further detail for each case in Supplementary Table S1.

We scored differentiation along mesenchymal lineages in each tumor using our convergence metric and ordered cases by the proportion of converged nuclei (Fig. 3C). This analysis corroborated our initial observation of a higher proportion of converged cells in high-risk cases: fusion-positive; lethal, fusion-negative; and *MYOD1*-mutated (*P* > 2.7 × 10^−5^, two-sided Mann–Whitney U test). Similarly, when we assessed the expression of the high-risk transcriptome (genes derived from the high-risk cluster in Fig. 2F), we found a significantly increased expression of high-risk genes in lethal, fusion-negative cases and *MYOD1*-mutated tumors (lethal and nonlethal disease; Fig. 3D; *P* < 2.2 × 10^−16^, two-sided Wilcoxon rank-sum test).

Next, we examined published transcriptomic data [single-cell (13), single-nuclei (10, 11), and bulk mRNA (6)] derived from RMS primary tumors as an orthogonal validation of our finding. Although detailed clinical outcomes for these cases were not available, we could subdivide tumors by fusion status and *MYOD1* mutation status. If our proposition of transcriptional convergence of high-risk RMS were true, we would expect fusion-positive and *MYOD1*-mutated tumors (which carry an unfavorable outcome) to be enriched for transcriptional features of high-risk cells. First, we applied our convergence metric to show that prognostically unfavorable fusion-positive tumors were significantly more converged than fusion-negative tumors (Fig. 3E; *P* < 2.2 × 10^−16^; two-sided Wilcoxon rank-sum test). We then examined each sample for the expression of high-risk changes, which we defined as genes that show increased expression prevalence in high-risk RMS clusters (as shown in Fig. 2F). We found significantly increased high-risk mRNA levels in fusion-positive tumors (*P* < 2.2 × 10^−16^; two-sided Wilcoxon rank-sum test) and elevated expression in *MYOD1*-mutated tumors from external single-cell and single-nuclei datasets and from external bulk mRNA datasets (Fig. 3F and G). Overall, these orthogonal lines of evidence in different cohorts corroborate our proposition of a convergent high-risk transcriptome that encompasses fusion-positive and lethal, fusion-negative tumors as well as RMS with prognostic unfavorable *MYOD1* mutation.

### Validation by spatial transcriptomics

**Figure 4.**
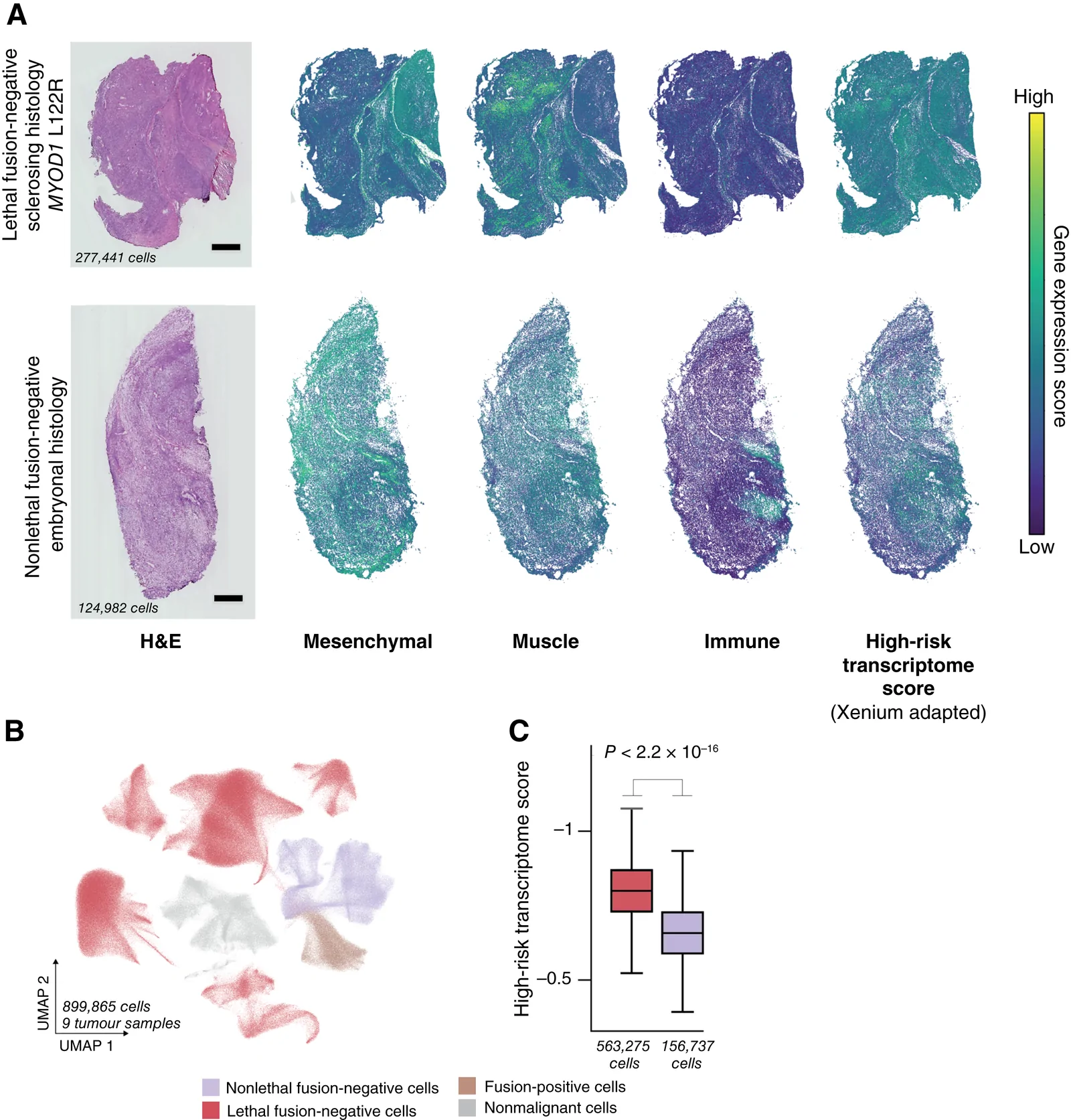
Spatial transcriptomic profiling confirms high-risk features and uncovers immune neighborhoods. **A,** Representative H&E images illustrate the tissue morphology of one lethal (red) and one surviving (purple) fusion-negative case. The scale bar represents 1,000 μm. Corresponding Xenium spatial transcriptomic scatter plots display cell-level expression patterns based on gene module scoring. Spatially variable genes were used to define three transcriptomic modules: muscle, mesenchymal, and immune. Cells were assigned module scores and visualized spatially to reveal regional transcriptomic heterogeneity. The Xenium high-risk score is also mapped across the tissue. Notably, the fusion-negative surviving case demonstrates a prominent localized immune-enriched region (“immune neighborhood”). **B,** UMAP plot shows the clustering of individual Xenium-extracted cells based on gene expression. Cells are colored by clinical subgroups: fusion-positive, fusion-negative alive, and lethal fusion-negative. **C,** Box plots show the distribution of Xenium high-risk scores for individual cells from FN lethal and FN surviving cases. Each box represents the interquartile range (IQR; 25th–75th percentile), the horizontal line within the box denotes the median, and whiskers extend to 1.5 times the IQR or the most extreme data point within that range. A two-sided Mann–Whitney U test was used to compare groups; a significant difference in module score distribution was observed.

We next used Xenium spatial transcriptomics, a single-cell assay profiling 5,000 transcripts, to validate the transcriptional convergence of high-risk cells in nine RMS specimens representing fusion-positive tumors, lethal fusion-negative tumors, and fusion-negative tumors with favorable outcome. One of the most striking findings was the spatially distinct expression of key mesenchymal and myogenic lineage markers within individual tumors. Using Moran I spatial autocorrelation analysis, we identified statistically significant regional enrichment of mesenchymal lineage markers such as *COL2A1*, *COL4A1/2*, and *POSTN* and spatially segregated expression of mature skeletal muscle markers, including *RYR1* and *TNNT2*, in three of nine cases evaluated (Fig. 4A; Supplementary Table S4, Moran I > 0.3 and FDR-adjusted *P* < 0.05). Spatial profiling for every case can be seen in Supplementary Fig. S3. These spatial expression patterns suggest that different regions within the same tumor exhibit distinct stages of myogenic differentiation, underscoring regionally organized transcriptional variability in lineage progression. Furthermore, high-risk transcriptional programs were not randomly distributed but formed spatially enriched regions, consistent with coordinated high-risk niches within the tumor (Supplementary Table S4). Notably, we also detected densely organized aggregates of T lymphocytes and signals of immune activation nested within the tumor (Supplementary Fig. S4A).

To interrogate RMS for evidence of transcriptional convergence, we mapped individual transcripts to spatially resolved cells, performed clustering, and annotated cell types accordingly (Fig. 4B; see Materials and Methods). We then applied the “high-risk” score, derived in Fig. 2, to score individual cells based on expression of 30 genes from the 89-gene signature that were represented in the Xenium 5K panel. This allowed us to spatially localize high-risk transcriptional programs within the tumor architecture. Overall, we found that cells within lethal fusion-negative and fusion-positive tumors exhibited significantly higher high-risk scores compared with those from fusion-negative survivors, consistent with our previous single-cell, multiome, and bulk RNA-seq analyses (Fig. 4C; Supplementary Fig. S4B, two-sided Mann–Whitney U test, *P* < 2.2 × 10^−16^). Decomposition of the high-risk signal into the fraction of high-risk–positive cells and their per-cell expression intensity revealed that this difference is driven predominantly by an increased proportion of high-risk–positive tumor cells, with more modest differences in per-cell expression intensity (Supplementary Fig. S4C). This spatial validation reinforces the robustness of the high-risk transcriptional program and reveals its localization within specific tumor niches.

### The nature of the convergent, high-risk transcriptome

Our analyses by different molecular assays and in different cohorts show that tumors of some children with lethal, fusion-negative disease converge transcriptionally with fusion-positive tumors. It is therefore possible that lethal, fusion-negative cells assume a fusion-positive cancer cell state, which the strong expression of mature muscle signals in both sets of tumors may suggest. Or, the convergent, high-risk transcriptome may represent different or additional features beyond the *FOXO1* fusion transcriptome.

**Figure 5.**
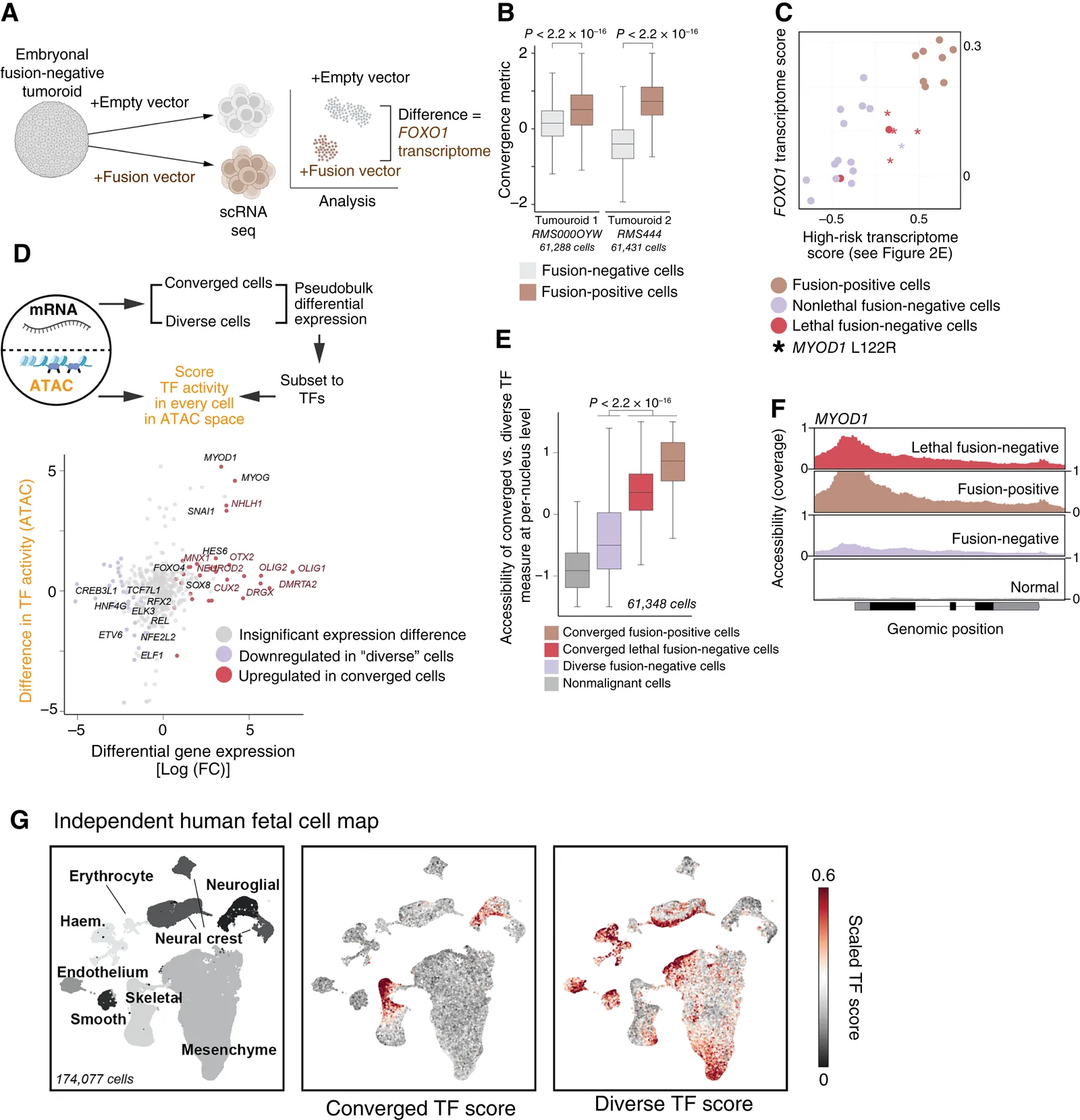
Characterization and functional validation of converged cells. **A,** Schematic illustrating the experimental workflow for RMS tumoroid perturbation models. Two RMS tumoroid lines (RMS000OYW and RMS444) derived from embryonal, fusion-negative tumors were experimentally induced with the *PAX3*–*FOXO1* fusion gene and subjected to scRNA-seq. Text annotation describes that we define the FOXO1 fusion transcriptome (or module) based on what is upregulated in cells that were induced with the fusion (brown), compared with control fusion-negative RMS cells (light gray). BioRender was used to generate some components of this figure. **B,** Boxplots showing the convergence metric across fusion-positive (brown) and fusion-negative (light gray) cells from two tumoroid models (see Materials and Methods). **C,** A scatter plot showing scores for the high-risk module (Supplementary Table S3) against the fusion module (Supplementary Table S5), averaged across RMS cells per patient in the extension cohort, colored by whether the tumor of a patient is fusion-positive (brown), fusion-negative (purple), or fusion-negative and died of their disease (red). Dots are shaped as an asterisk if the patient harbors *MYOD1*-mutant cells. The high-risk module is as defined in Fig. 2, and the fusion module was derived from the fusion experiments in (**A**; See Materials and Methods). **D,** First, a schematic illustrating the analytic workflow for the snATAC-seq data in the extension cohort. We use the gene expression component of the data to obtain differentially expressed genes between converged and diverse cells. We then subset differentially expressed genes to TFs and calculate per-cell scores to assess chromatin availability of the motifs for these TFs. BioRender was used to generate some components of this schematic. The bottom half of the panel is a scatter plot summarizing both the differential gene expression (*x*-axis) and difference in TF motif availability (*y*-axis) between converged and diverse cells, where every dot represents a TF. Dots are colored if they are significantly differentially expressed in either converged cells (red) or diverse cells (purple) and left gray if nonsignificant. TFs upregulated in converged cells have a positive log FC. The *y*-axis shows the average chromVAR score in converged cells subtracted by the average chromVAR score in diverse cells (see Materials and Methods). TF names are colored red if they are annotated as being associated with a neural lineage. **E,** Boxplots showing the difference in the averaged TF motif accessibility of converged and diverse TFs (i.e., converged TF average − diverse TF average) across normal cells (gray), diverse cells from fusion-negative tumors (purple), and converged cells from fusion-positive (red) and lethal fusion-negative tumors (brown). **F,** Coverage tracks showing chromatin accessibility for converged nuclei from lethal fusion-negative cases (red), fusion-positive nuclei (brown), diverse fusion-negative nuclei (purple), and nonmalignant nuclei (gray) at the *MYOD1* locus. **G,** A UMAP of 174,077 cells from the developmental atlas of the human fetal spine, colored by broad cell type provided by the authors (left), converged TF score (middle), diverse TF score (right). The TF scores are calculated in scanpy and represent an aggregated score of the TFs defined by the analysis in **D**. *P* values were calculated using a two-sided Wilcoxon rank-sum test. **A,** Created in BioRender. Anderson, N. (2026) https://BioRender.com/h758tn5; **D,** Created in BioRender. Anderson, N. (2026) https://BioRender.com/aw07wgo.

We addressed this question by comparing transcriptional effects of the *FOXO1* fusion with fusion-negative high-risk transcriptomes. First, we distilled the *FOXO1* fusion transcriptome from two RMS tumoroid models derived from embryonal fusion-negative tumors (31). We lentivirally transduced fusion-negative tumoroid cultures with vectors to deliver a *FOXO1* fusion construct and compared, by single-cell mRNA sequencing, converted, now fusion-positive cells with cells derived from cultures transduced with a vector expressing a control coding sequence (CDS) (see Materials and Methods; Fig. 5A). This experiment confirmed that the gene fusion is capable of inducing the converged cell state (Fig. 5B; *P* < 2.2 × 10^−16^, two-sided Wilcoxon rank-sum test). Next, we assessed whether the downstream transcriptome of the gene fusion fully accounted for the transcriptional convergence. To this end, we compared the *in vitro*-derived *FOXO1* transcriptome with the high-risk transcriptome as defined from the discovery cohort (genes elevated in the high-risk cluster vs. fusion-negative cells with favorable outcome; Fig. 2F). This analysis showed that the convergence of high-risk tumors (lethal fusion-negative and *MYOD1*-mutated) encompasses direct transcriptional changes induced by the *FOXO1* gene fusion (Supplementary Table S5) and an additional component of mRNA changes not accounted for by the fusion (Fig. 5C). Even when we scored with previously published *FOXO1* fusion gene signatures (44), the transcriptomic effects of the fusion did not fully capture convergence of high-risk tumors (Supplementary Fig. S5).

To annotate the high-risk transcriptome qualitatively, we deployed a further orthogonal assay, single-nucleus ATAC sequencing, which we applied to the extension cohort. To define TFs that were upregulated in converged nuclei, we performed a differential gene expression analysis between high-risk converged nuclei and diverse nuclei from children with favorable outcomes (Fig. 5D). We then quantified the accessibility of these differentially expressed TFs using chromVAR (20), a computational framework that infers TF activity by scoring cells based on chromatin accessibility at TF binding motifs (Supplementary Table S6). We found that accessibility and expression were largely concordant, suggesting that TFs with more accessible binding sites were also expressed and likely active (Fig. 5D and E). Examining the identity of TFs with higher activity in converged cells, we find known muscle-lineage TFs such as *MYOD1* (Fig. 5F) and *MYOG*, in addition to other TFs (e.g., *OLIG2*, *CUX2*, *DMRTA2*, and *MNX1*) that are characteristic of neural lineages (Supplementary Figs. S6 and S7). Muscle and neural markers coincided within cells and nuclei, indicating that these differentiation states commonly exist within the same cell and do not primarily represent distinct cell populations (Supplementary Fig. S7A and S7B).

We then sought to contextualize these transcriptional states using an independent human fetal reference (*n* = 174,077 cells; ref. 45). Importantly, this reference is entirely distinct from the reference used to derive the convergence metric (Fig. 2), ensuring that our analysis remains non-circular. We scored normal fetal cells with TFs of converged cells (converged TF score) and TFs of cells with diverse differentiation (diverse TF score) and found that converged TFs were largely specific to muscle and neural lineages (Fig. 5G; Supplementary Fig. S7C and S7D; Supplementary Table S6). To further evaluate the extent of neural gene expression encompassed by the converged cell state, we inspected each gene of the high-risk transcriptome and found that beyond genes related to muscle development (∼23%), at least 20% of genes define neural lineages or are neural-associated, including *MYCN* and *NOS1* (Supplementary Table S3). A GO enrichment analysis of the high-risk transcriptome, however, suggests that the dominant signal in this cell state is muscle and that neural gene expression is a minor component (Supplementary Fig. S8A). This was supported by the increased expression of our high-risk score in differentiated and neuronal cell populations defined by Danielli and colleagues (Supplementary Fig. S8B; ref. 14). We further investigated the nature of the high-risk transcriptome by comparing it with the existing gene signatures in the literature (Supplementary Table S7; refs. 11, 12). No overlap was statistically significant, including neuronal-like signatures described in fusion-positive cells, suggesting that the high-risk transcriptome reflects a distinct state that unifies fusion-positive and lethal fusion-negative tumors. These findings highlight the complex and overlapping nature of the high-risk convergent cell state, which may contribute to the aggressive behavior observed in these cancers.

### Distinct targets for immunotherapy in high-risk cells

**Figure 6.**
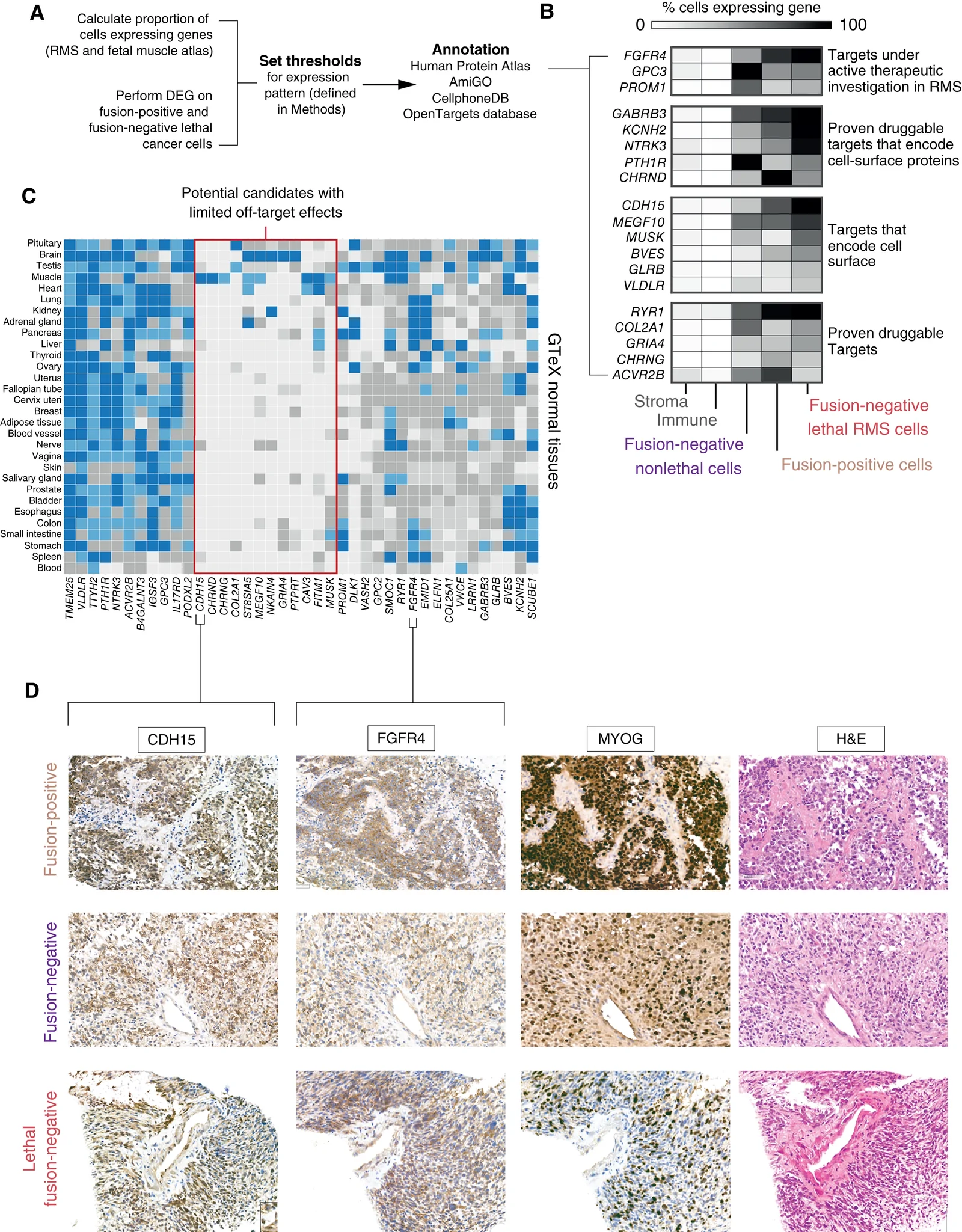
Prioritization of therapeutic targets in high-risk RMS. **A,** Flowchart outlines the analytic workflow used to identify potential therapeutic targets from scRNA-seq data. The pipeline integrates transcriptomic profiling, tissue specificity filters, and druggability annotations to systematically triage candidate genes into tiered categories based on predefined criteria. **B,** Heatmaps display the proportion of cells expressing candidate genes across samples, grouped into three tiers: tier 0, known RMS targets; tier 1, genes that are highly and selectively expressed in RMS tissue, also present in fetal muscle, and predicted to be located on the cell surface; and tier 2, genes satisfying any one of the tier 1 criteria. Within tiers 1 and 2, genes were further prioritized if they overlapped with entries in the Open Targets platform, indicating existing evidence of druggability. Genes are ranked by the proportion of expressing cells within the fusion-negative lethal group, highlighting potential targets enriched in aggressive disease. **C,** Heatmap illustrates the mean logCPM expression levels of tier 1 and tier 2 candidate genes across a range of normal human tissues from the GTEx database. Expression values were categorized into five discrete bins: extremely low (≤0), very low (>0 to ≤1), low (>1 to ≤3), moderate (>3 to ≤4), and high (>4). Both genes and tissues are hierarchically clustered. Genes exhibiting highly restricted expression across normal tissues form a distinct clade, emphasizing their potential therapeutic selectivity and potential to minimize expected off-target effects. **D,** IHC validation of candidate cell-surface targets in representative RMS subtypes. Representative IHC staining for CDH15 and FGFR4 in three primary RMS cases, shown alongside myogenin (MYOG) and H&E staining for histologic reference. Cases include one fusion-positive tumor, one lethal fusion-negative tumor, and one nonlethal fusion-negative tumor. These images illustrate the extent and pattern of tumor-cell staining for candidate cell-surface markers across clinically distinct RMS groups.

With immunotherapy actively being developed as a treatment strategy for RMS, we next investigated whether lethal fusion-negative and fusion-positive RMS harbored overlapping or distinct molecular features that could be targeted therapeutically. Particularly attractive in this context are fetal developmental antigens, the physiologic expression of which is confined to fetal life. We developed an analytic framework for target discovery, built on our previously published approach (Fig. 6A; see Materials and Methods; ref. 46). In brief, we conducted a broad analysis to identify transcripts that are specific to high-risk RMS cells and then searched for fetal transcripts that are silenced in most postnatal tissues, thus making them attractive candidates for targeted therapy. This approach identified a variety of putative targets, which we subdivided into different priority tiers based on their biological relevance, druggability, and potential as therapeutic targets, as defined in Fig. 6B (see Supplementary Table S8 for the full list of targets). We identified widely pursued targets in RMS, including *GPC3* (47) and *FGFR4* (48), which we found to be preferentially expressed by nonlethal and lethal cells, respectively. Of note, our unbiased target identification revealed neural genes (e.g., *GABRB3* and *KCNH2*), which were particularly elevated in fusion-positive and in lethal, fusion-negative disease, further substantiating a neural component of the convergent, high-risk RMS transcriptome. We assessed the expression of these putative targets in normal, postnatal tissues [GTEx data (37)], which indicated a favorable therapeutic profile that may reflect limited off-target effects (Fig. 6C; Supplementary Fig. S9).

To provide orthogonal validation of candidate surface targets identified by single-cell transcriptomic analysis, we assessed selected prioritized antigens in RMS by IHC and flow cytometry. FGFR4 was included as a positive control because it has previously been described as an immunotherapeutic target in RMS (48), whereas CDH15 and cholinergic receptor subunits were prioritized on the basis of tumor-restricted developmental expression. IHC across 10 RMS specimens (six fusion-negative and four fusion-positive) showed positive FGFR4 staining in 9 of 10 cases and positive CDH15 staining in 9 of 10 cases (Fig. 6D). We also considered the fetal nicotinic acetylcholine receptor program, in which the γ subunit encoded by *CHRNG* is replaced by the epsilon subunit in postnatal skeletal muscle, suggesting that persistence of *CHRNG* expression may represent an oncofetal and tumor-selective feature. Although CHRNG could not be reliably assessed by IHC because of a lack of suitable antibodies, flow cytometry detected surface expression in four of five RMS cell lines and in all three primary tumor samples examined (Supplementary Fig. S10). Together, these findings support the protein-level expression of prioritized targets in RMS and highlight the fetal isoform of the fetal acetylcholine receptor, CHRNG, as a candidate for antigen-directed therapy despite relatively low surface abundance, particularly given its developmental restriction and the capacity of engineered cell therapies to target low-density antigens.

## Discussion

RMS may be incurable for a variety of reasons, such as disseminated disease at diagnosis or aggressive cancer cells that render even localized tumors difficult to eradicate. The principal marker of aggressive cancer cells is the *FOXO1* fusion, which is closely linked to a mature muscle cell state in most tumors. We examined lethal tumors that are fusion-negative and found that they transcriptionally converged with fusion-positive RMS cells. This finding indicates that an overarching cell state of aggressive disease exists in RMS, and comparison with existing signatures suggests that it has not been captured by previous studies. The state overlaps partially with the *FOXO1* transcriptome and exhibits distinctive features, representing a hybrid neural and muscle state in human developmental terms. This hybrid identity is supported by previous reports of aberrant expression of neural and neuroendocrine markers in RMS (49). Convergence is largely, but not fully, explained by molecular mimicry of the gene fusion. This suggests that aggressive behavior in RMS may be better understood, at least in part, as a transcriptional cell state rather than solely as a consequence of a specific initiating genotype. In this framework, fusion-positive disease and a subset of clinically aggressive fusion-negative tumors may reach a common downstream program through distinct upstream molecular routes. This provides a way to reconcile why some fusion-negative tumors behave more aggressively than would be expected from their conventional molecular classification.

Our findings build on multiple lines of evidence derived from different cohorts, including external data and from a variety of assays. It is therefore unlikely that the signal we identified is driven by technological or patient biases. What enabled our discovery was close collaborations with clinical teams to accurately capture disease phenotypes and patient outcomes, which guided our analyses. However, patient outcomes also represent a potential confounder in our analysis, as we cannot exclude that children with good outcomes will relapse in the future. Even so, given the scale of the experiment and extensive validation of the signal, it is unlikely that our findings will fundamentally change. Furthermore, with a median follow-up time of 44 months, we have allowed enough time for most relapses to occur, given that RMS typically recurs within 36 months from diagnosis (50).

A key question our findings raise is what underpins the convergent, high-risk cell state, which did not associate with any histologic pattern or somatic variant, apart from an enrichment of *MYOD1* L122R mutations. This variant has been linked to poor outcomes in RMS, and it is remarkable that our findings show transcriptional convergence between *MYOD1*-mutant tumors and fusion-positive RMS. It is possible that some of the driver (cancer-causing) mutations we identified in fusion-negative lethal disease [e.g., a rare *NOTCH3* fusion or other recently described gene fusions (51, 52)] may emerge as the culprit in the future as our understanding of the RMS genome extends. Accordingly, what unifies lethal disease may be low-frequency, high-risk somatic mutations that converge in a common cell state. To date, the largest studies of RMS genomes encompass no more than 44 tumors (6), and studies of several thousand genomes from meticulously annotated cohorts will be required to test this hypothesis. Alternatively, the high-risk cell state may be driven by epigenetic variation or by chance.

We would suggest that apart from their biological significance—the proposition of an overarching cancer cell state of aggressive disease—our findings have potential clinical significance. By examining larger cohorts prospectively, it may be possible to validate gene signatures of aggressive disease that could be utilized for risk stratification. This includes identifying apparently fusion-negative tumors that nonetheless harbor a high-risk transcriptional program and may therefore warrant closer monitoring or intensified therapy. Our results prioritize aggressive fusion-negative outlier cases for deeper genomic, epigenomic, and functional studies, as these tumors may reveal alternative routes into the same lethal cell state. Importantly, although the 89-gene signature reproducibly identifies a high-risk RMS cell state, our study does not provide direct functional evidence that this program drives aggressive disease. We therefore view the signature as defining a convergent transcriptional state associated with high-risk RMS, rather than establishing a causal mechanism. Similarly, although our analysis nominates candidate antigens associated with this state, their prioritization for target discovery will require further validation of expression across normal tissues, particularly in pediatric contexts.

In the wider context of emerging cancer cell atlases, the significance of our findings is to reveal a cancer cell state, as represented in mRNA, that transcends conventional prognostic boundaries, even in a cancer type that is as molecularly and clinically thoroughly characterized as RMS. Our approach of focusing quantitative molecular analyses on disease patterns may reveal similar findings in other cancer types.

## Supplementary Material

Supplementary Figure 1 Cell type annotation of the ‘Discovery’ Cohort.

Supplementary Figure 2 High-risk transcriptome scores by risk category.

Supplementary Figure 3 Xenium spatial maps across RMS specimens.

Supplementary Figure 4 Xenium immune response signals and high-risk score summary across RMS samples.

Supplementary Figure 5 Fusion-associated signatures do not capture the high-risk transcriptome.

Supplementary Figure 6 Chromatin accessibility at upregulated transcription factor loci in converged cells.

Supplementary Figure 7 Co-expression of converged cell transcription factors from muscle and neural lineages in RMS tumours.

Supplementary Figure 8 Gene Ontology enrichment of the high-risk transcriptome.

Supplementary Figure 9 Cellular Prevalence and Postnatal Tissue Expression of Prioritized RMS Targets.

Supplementary Figure 10 Detection of fAChR in RMS cell lines and RMS tumour tissue.

Supplementary Table 1 Supplementary Table 1

Supplementary Table 2 Supplementary Table 2

Supplementary Table 3 Supplementary Table 3

Supplementary Table 4 Supplementary Table 4

Supplementary Table 5 Supplementary Table 5

Supplementary Table 6 Supplementary Table 6

Supplementary Table 7 Supplementary Table 7

Supplementary Table 8 Supplementary Table 8

Supplementary Table 9 Supplementary Table 9

## Data Availability

Sequencing data for the discovery and extension cohorts (DNA, bulk mRNA, single-cell mRNA, and single-nuclei mRNA sequencing) are available through the European Genome-Phenome Archive. Accession codes and descriptions for data generated in this study can be found in Supplementary Table S9. Processed and raw counts for each of the single-cell and single-nuclei mRNA sequencing datasets (discovery and extension cohorts) are available for visualization (click “explore”) and download (click “download”) through CELLxGENE: https://cellxgene.cziscience.com/collections/67d26b1d-2b78-4489-ac5e-0f33909a76cb. Raw and processed Xenium data have been uploaded to Zenodo and can be accessed https://zenodo.org/records/16785315 (53). All other raw data are available upon request to the corresponding author.

## Code Availability

No previously unreported custom computer code or algorithms were used to generate results that are reported in this article. Code used to analyze data and produce figures for this study are available in the Behjati Lab repository: https://github.com/BehjatiLab/RMS-lethal-sc-convergence.
