# Supplementary material for: High-Risk Rhabdomyosarcomas Feature a Convergent Cell State
Source: Cancer Res. Author manuscript; Available in PMC 2026 Oct 10. (PMC13631504; doi:10.1158/0008-5472.CAN-25-4403)
Supplement: Supplementary file 1 [file EMS218232-supplement-Supplementary_Figure_1.pdf]

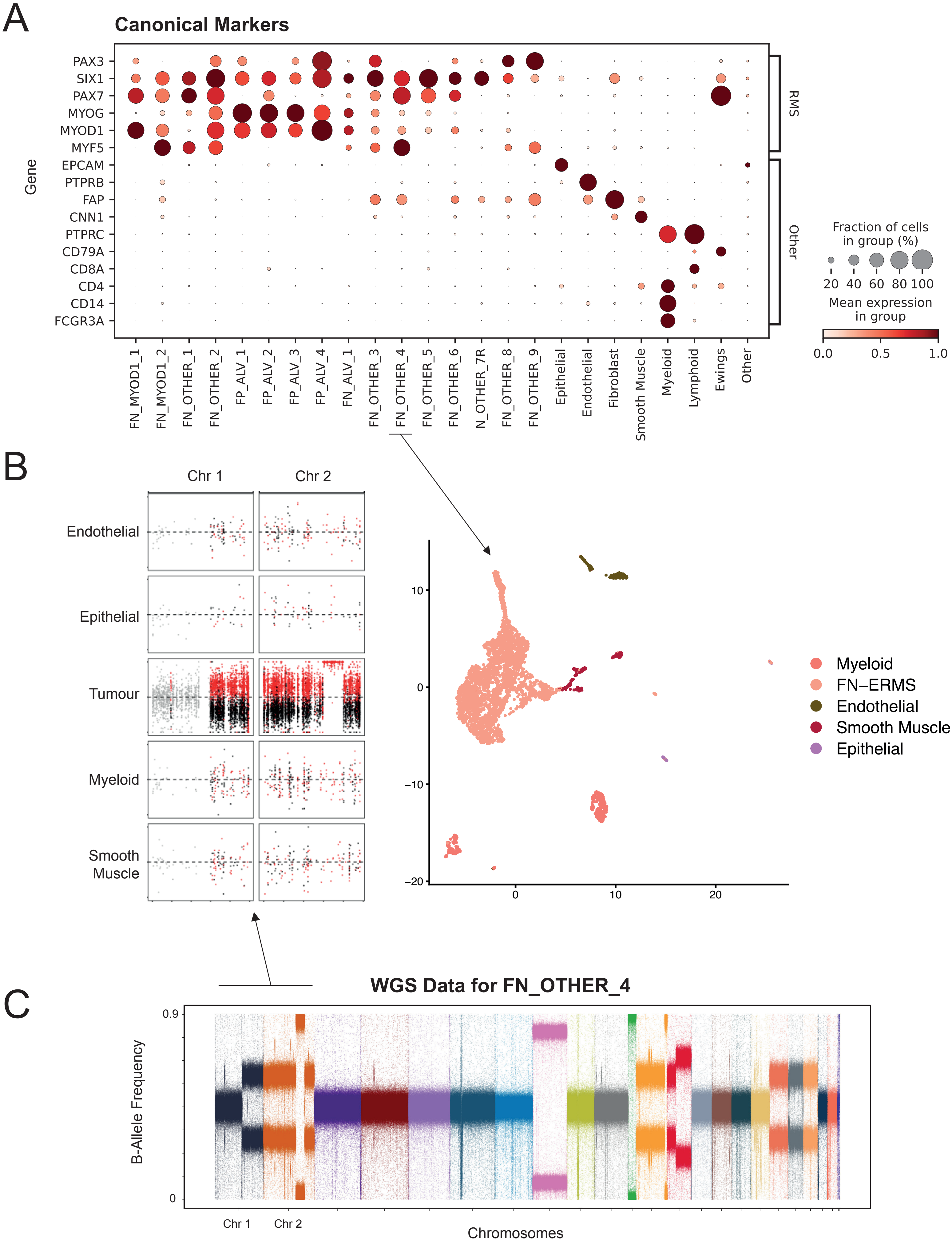


**Supplementary Figure 1: Cell type annotation of the 'Discovery' Cohort.** Cell type annotation is based on **(A)** canonical markers and **(B)** somatic copy number variation. **(C)** Copy-number plots illustrate reference whole-genome copy number calls. B-allele frequencies across all chromosomes are shown.
