# Supplementary material for: High-Risk Rhabdomyosarcomas Feature a Convergent Cell State
Source: Cancer Res. Author manuscript; Available in PMC 2026 Oct 10. (PMC13631504; doi:10.1158/0008-5472.CAN-25-4403)
Supplement: Supplementary file 2 [file EMS218232-supplement-Supplementary_Figure_10.pdf]

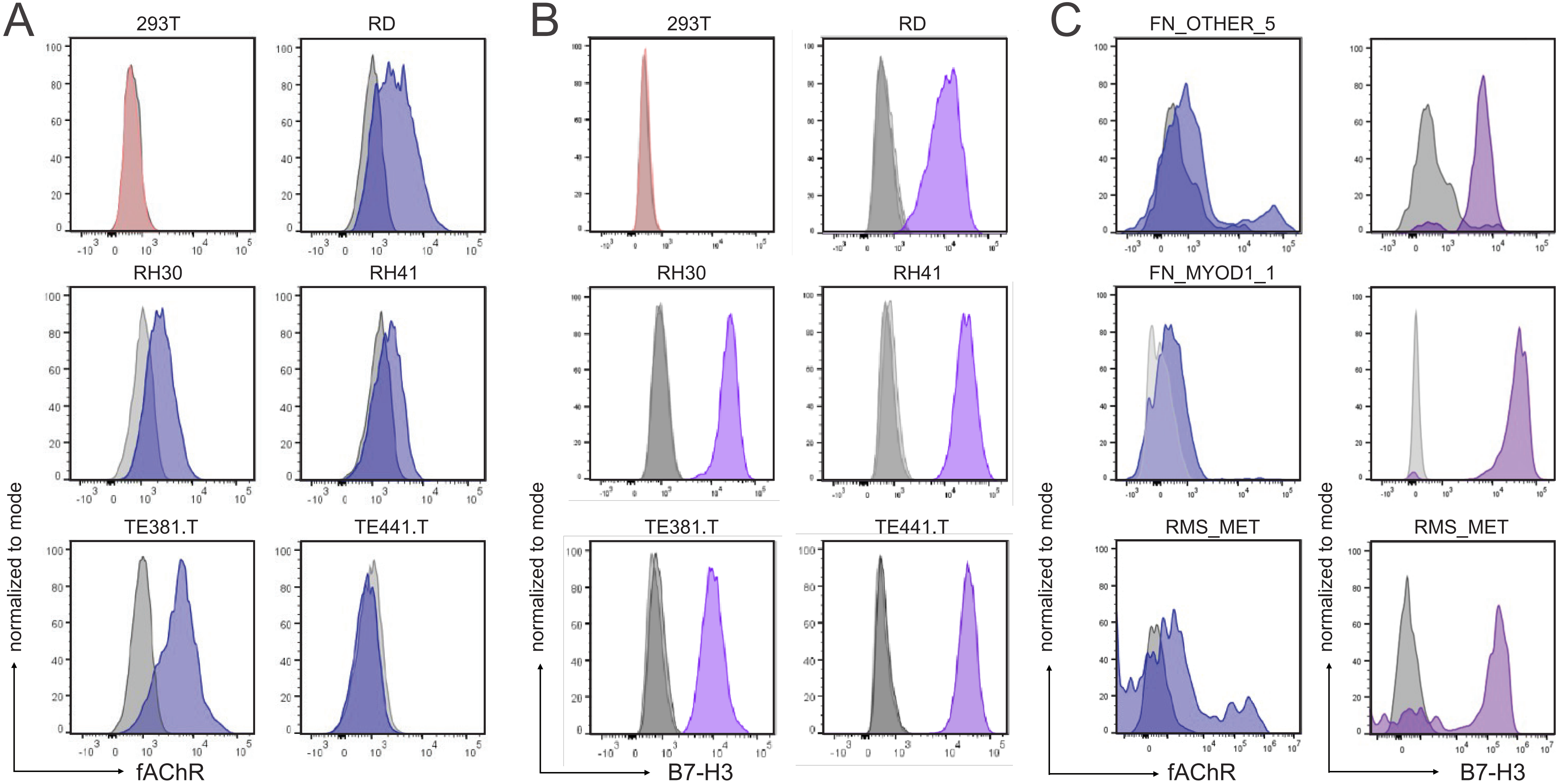

Supplementary Figure 10. Detection of fAChR in RMS cell lines and RMS tumour tissue. Shown is detection of fAChR (containing the CHRNG-encoded γ subunit **(A)** and described RMS target B7-H3 for reference **(B)** on a panel of RMS cell lines. Presence of fAChR is detected using mAb131 antibody and secondary anti-mouse IgG2a antibody. Grey indicates unstained/staining with secondary antibody only, while colours indicate staining for indicated markers. 293T and Jurkat were used as control cell lines not expressing indicated markers. **(C)** Shown is the expression of fAChR and B7-H3 on fresh RMS tumour tissue dissociated into single cell suspension for 3 different tumour samples. Tumour cells selected for assessment of target antigen expression were gated as single, live, CD3negCD45neg cells. Colours indicate full stain while grey indicates fluorescence minus one (FMO) staining control. Median fluorescence intensity (MFI) is shown on the X axis and counts normalised to mode is shown on the Y axis.
