# Supplementary material for: High-Risk Rhabdomyosarcomas Feature a Convergent Cell State
Source: Cancer Res. Author manuscript; Available in PMC 2026 Oct 10. (PMC13631504; doi:10.1158/0008-5472.CAN-25-4403)
Supplement: Supplementary file 3 [file EMS218232-supplement-Supplementary_Figure_2.pdf]

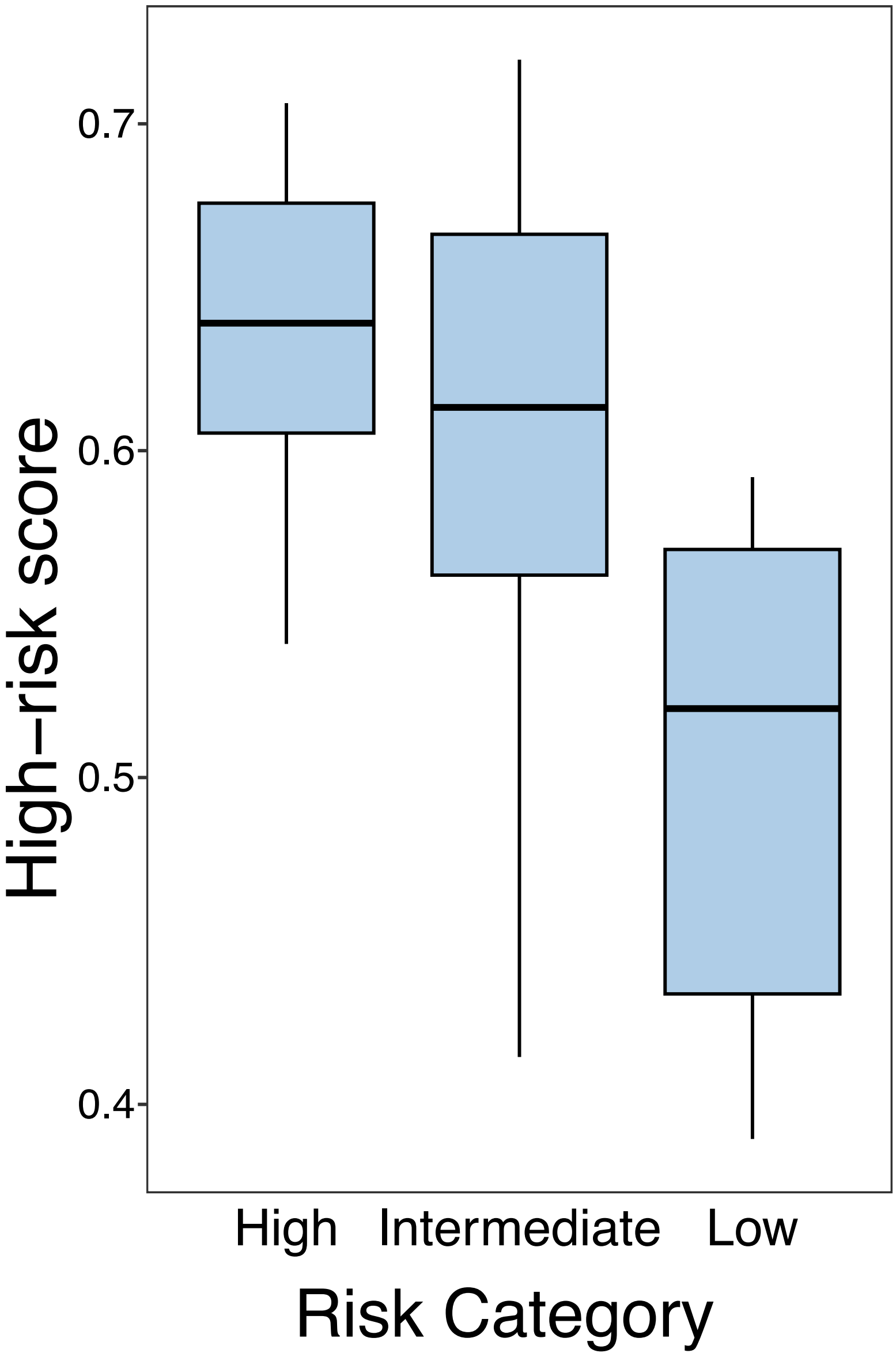

Supplementary Figure 2: High-risk transcriptome scores by risk category. Microarray samples from Williamson et al.43 scored with our high-risk transcriptome score. Each boxplot represents all samples in one of three risk groups defined by the paper. Risk categories (low, intermediate, high) from this cohort are not directly comparable to contemporary classifications, as they were primarily based on clinical and histopathological features, and did not incorporate molecular markers such as FOXO1 fusion status.
