# Supplementary material for: High-Risk Rhabdomyosarcomas Feature a Convergent Cell State
Source: Cancer Res. Author manuscript; Available in PMC 2026 Oct 10. (PMC13631504; doi:10.1158/0008-5472.CAN-25-4403)
Supplement: Supplementary file 4 [file EMS218232-supplement-Supplementary_Figure_3.pdf]

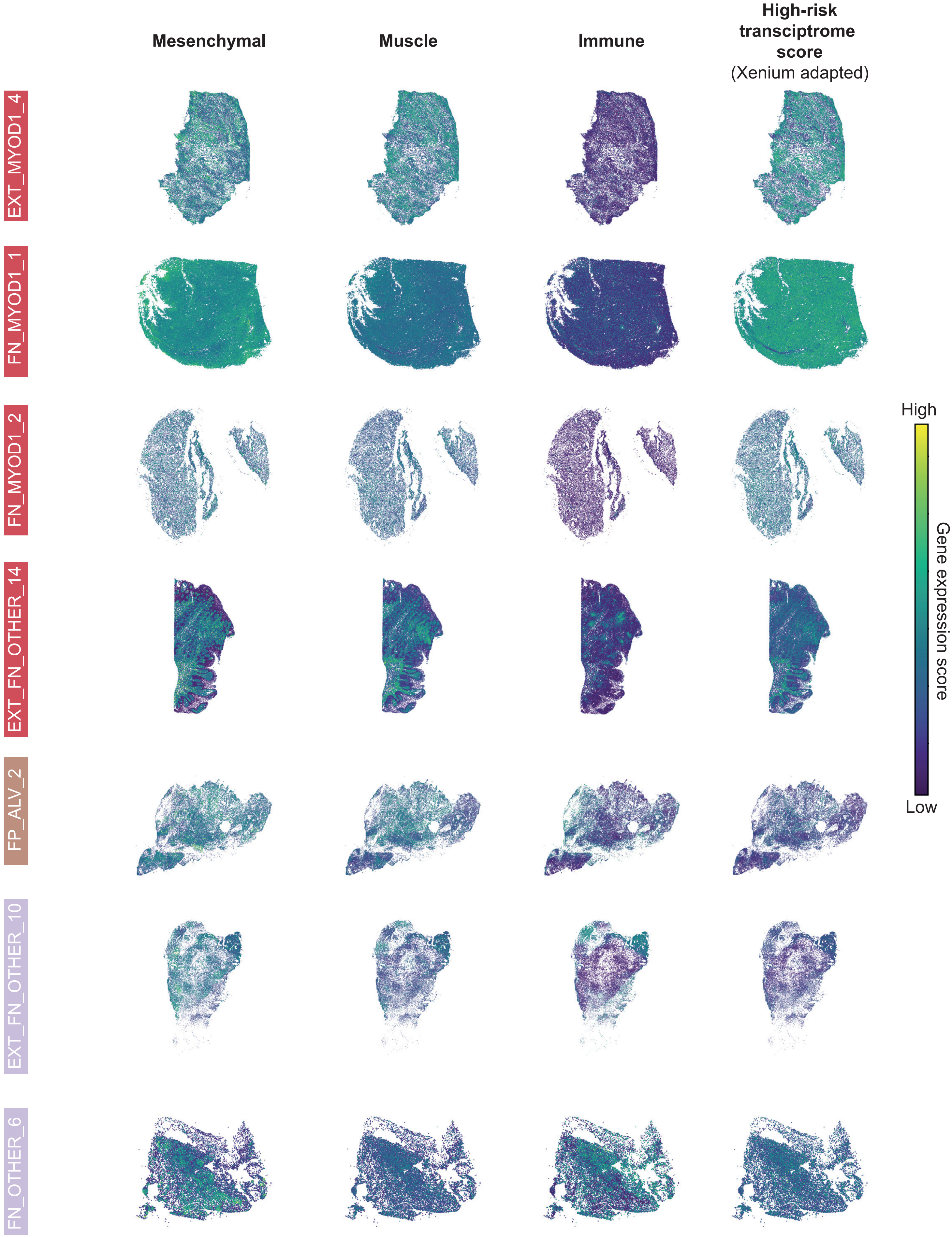

Supplementary Figure 3: Xenium spatial maps across RMS specimens. Spatial maps of the mesenchymal, immune, muscle, and Xenium-adapted high-risk scores for the seven RMS specimens not shown in the main figure. Each panel displays per-cell scores in tissue space, illustrating the local distribution of these programmes within the profiled section. Together with the representative cases in Figure 4, these data show the spatial heterogeneity of transcriptional states across the full Xenium cohort in this study.
