# Supplementary material for: High-Risk Rhabdomyosarcomas Feature a Convergent Cell State
Source: Cancer Res. Author manuscript; Available in PMC 2026 Oct 10. (PMC13631504; doi:10.1158/0008-5472.CAN-25-4403)
Supplement: Supplementary file 5 [file EMS218232-supplement-Supplementary_Figure_4.pdf]

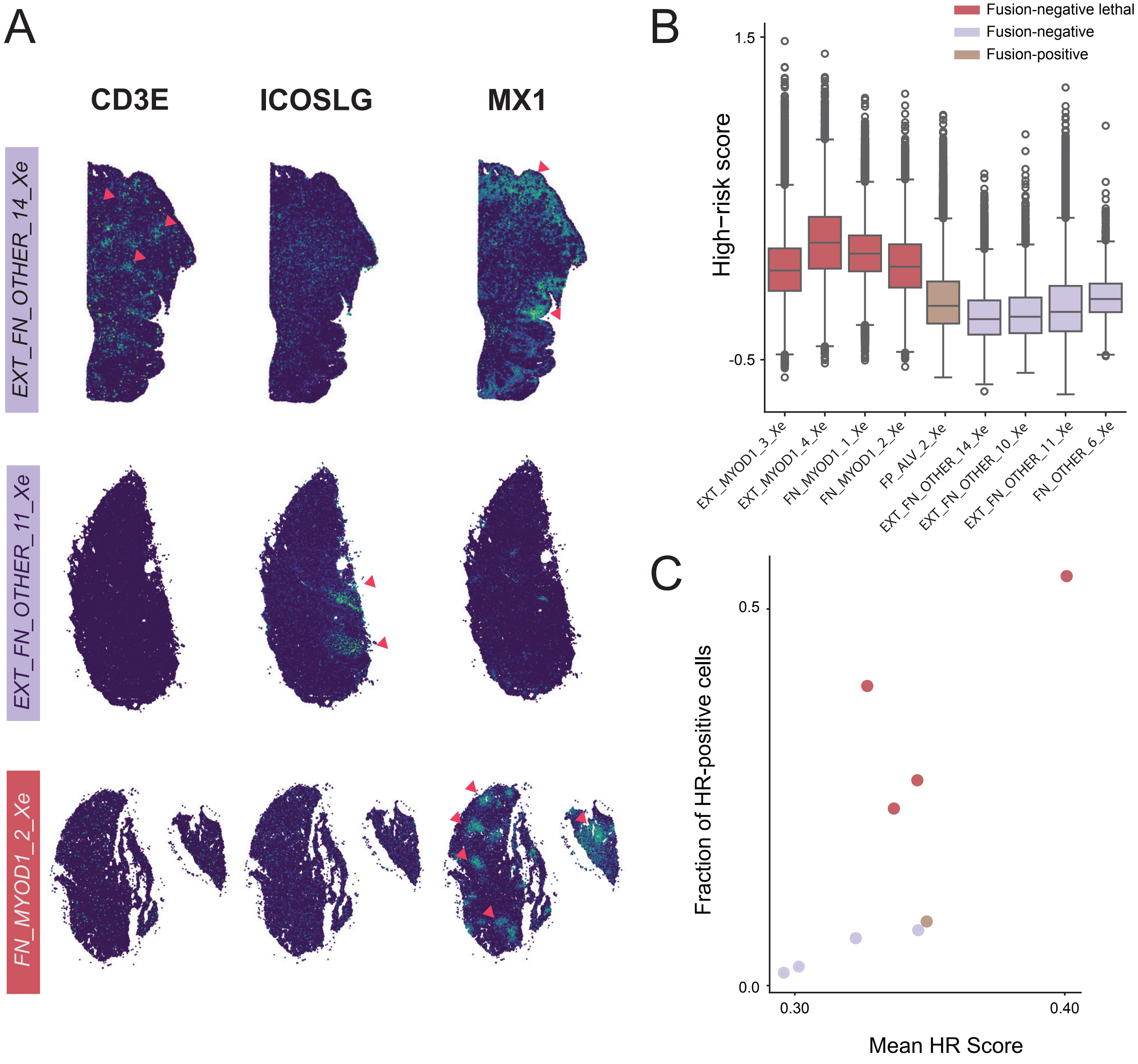

Supplementary Figure 4: Xenium immune response signals and high-risk score summary across RMS samples. (A) Spatial scatter plots of rhabdomyosarcoma tissue sections showing the distribution of T cells within the tumour microenvironment. Spots are colored by expression of a canonical T cell activation (CD3E), co-stimulation of T cells by antigen-presenting cells (ICOSLG) and antiviral defense via interferons (MX1), revealing densely organized intratumoral aggregates, as indicated by the red arrows. These immune cell clusters are spatially confined and suggest focal immune niches embedded within the tumour parenchyma. Data shown for representative cases. **(B)** Boxplots show high-risk transcriptomic scores for individual RMS samples, stratified by groupings. The y-axis represents xenium high-risk scores derived from spatial transcriptomic profiling. Each boxplot displays the interquartile range (IQR), with the box spanning the 25th to 75th percentiles and the horizontal line indicating the median. Outliers were removed for visualization clarity. Fusion-positive tumours and lethal fusion-negative lethal cases exhibit significantly higher high-risk scores compared to fusion-negative survivors. **(C)** Scatter plot shows the patient-level prevalence and intensity of the high-risk programme. High-risk-positive cells were defined as malignant cells with a high-risk score greater than 0.2. For each sample, we calculated the proportion of high-risk-positive cells, the mean high-risk score among those cells, and the total numbers of malignant and high-risk-positive cells.
