# Supplementary material for: High-Risk Rhabdomyosarcomas Feature a Convergent Cell State
Source: Cancer Res. Author manuscript; Available in PMC 2026 Oct 10. (PMC13631504; doi:10.1158/0008-5472.CAN-25-4403)
Supplement: Supplementary file 6 [file EMS218232-supplement-Supplementary_Figure_5.pdf]

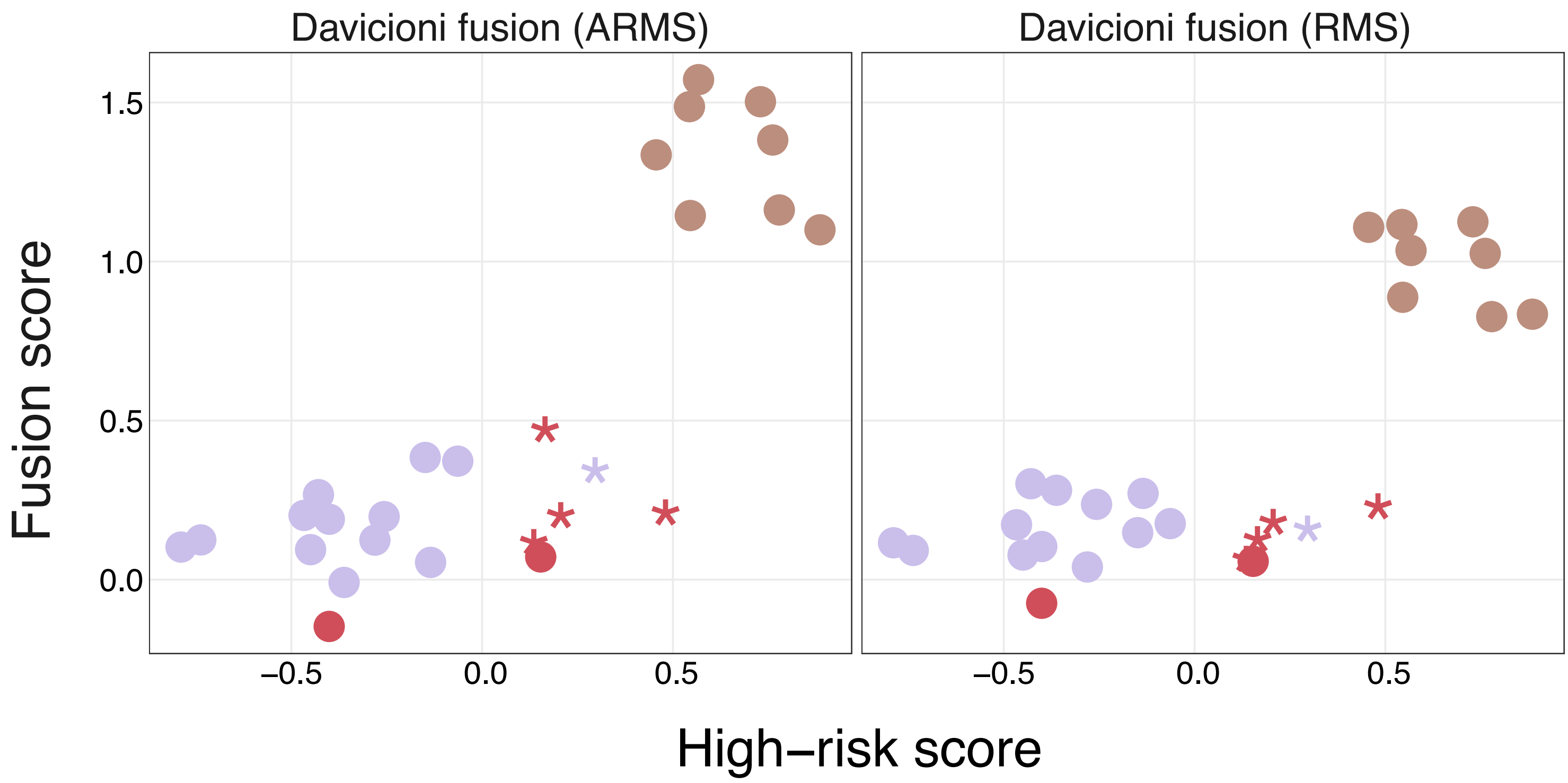

Supplementary Figure 5. Fusion-associated signatures do not capture the high-risk transcriptome. A scatter plot illustrating per-cell scores using published FOXO1-fusion modules. Each dot is the average score for each patient of the single-nuclei cohort. The x-axis shows scores for the high-risk transcriptome and the y-axis displays scores for the two Davicioni fusion modules: derived from comparing primary tumours (left) and cell lines (right). Signatures used here, "DAVICIONI_RHABDOMYOSARCOMA_PAX_FOXO1_FUSION_UP" and "DAVICIONI_PAX_FOXO1_SIGNATURE_IN_ARMS_UP", were downloaded from MSigDB. Colors: fusion-positive (brown), fusion-negative (purple), or fusion-negative and died of their disease (red). Dots are shaped as an asterisk if the patient harbours MYOD1-mutant cells.
