# Supplementary material for: High-Risk Rhabdomyosarcomas Feature a Convergent Cell State
Source: Cancer Res. Author manuscript; Available in PMC 2026 Oct 10. (PMC13631504; doi:10.1158/0008-5472.CAN-25-4403)
Supplement: Supplementary file 7 [file EMS218232-supplement-Supplementary_Figure_6.pdf]

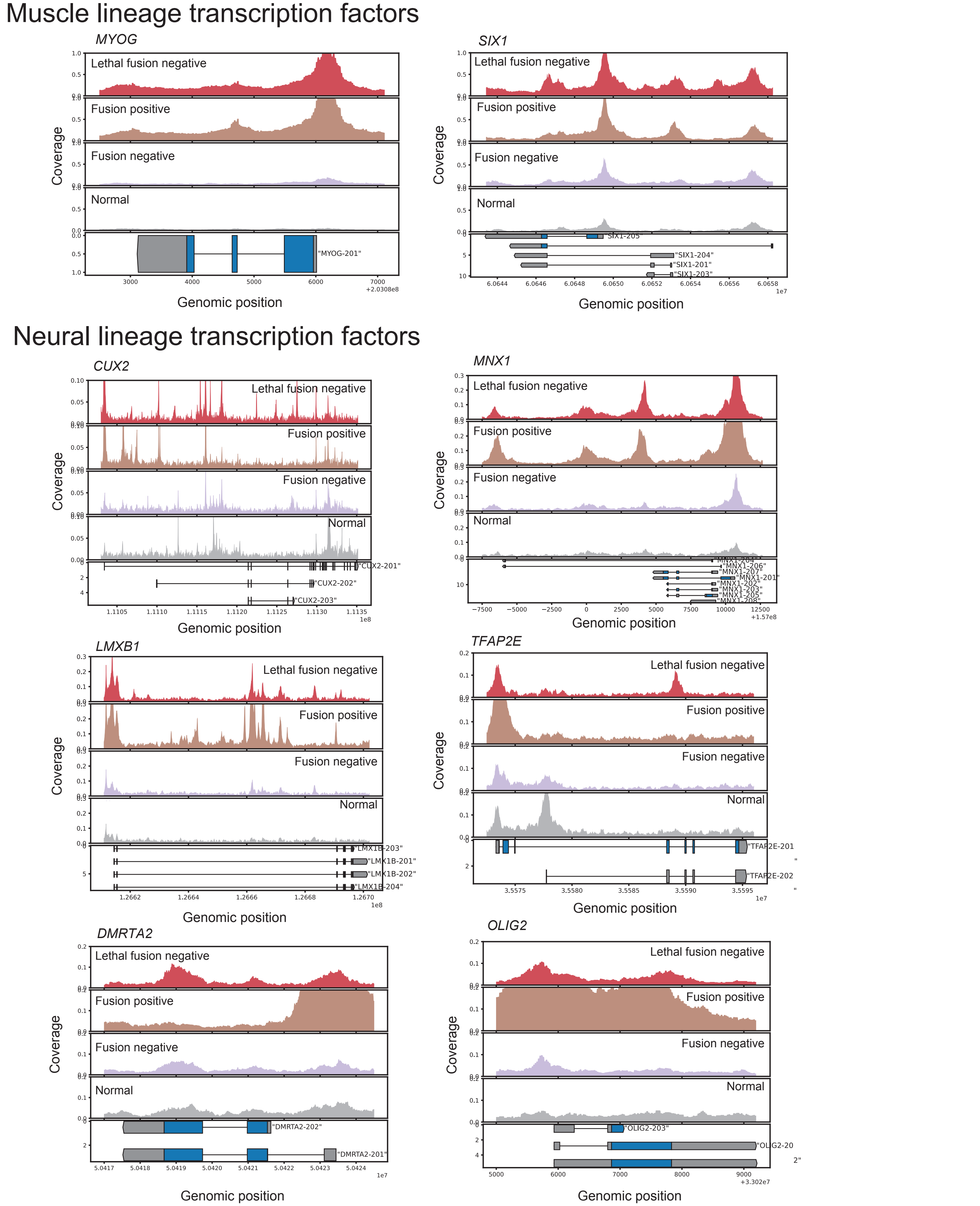

Supplementary Figure 6. Chromatin accessibility at upregulated transcription factor loci in converged cells. Single-nucleus ATAC-seq (snATAC-seq) coverage tracks from the extension cohort, showing chromatin accessibility at selected transcription factor (TF) gene loci that are significantly upregulated in converged nuclei. The targets of these transcription factors are also highly accessible in converged cells (Figure 5D). Tracks are aggregated per cell label: converged nuclei from lethal fusion-negative patients (red), converged nuclei from fusion-positive patients (brown), diverse nuclei from fusion-negative patients (purple), and normal immune and stromal nuclei (grey). Regions of high accessibility suggest transcription factor expression and support the differential expression analyses performed using the mRNA component of the data. Coverage and GTF tracks were generated using pyGenomeTracks (RRID:SCR_025312).
