# Supplementary material for: High-Risk Rhabdomyosarcomas Feature a Convergent Cell State
Source: Cancer Res. Author manuscript; Available in PMC 2026 Oct 10. (PMC13631504; doi:10.1158/0008-5472.CAN-25-4403)
Supplement: Supplementary file 8 [file EMS218232-supplement-Supplementary_Figure_7.pdf]

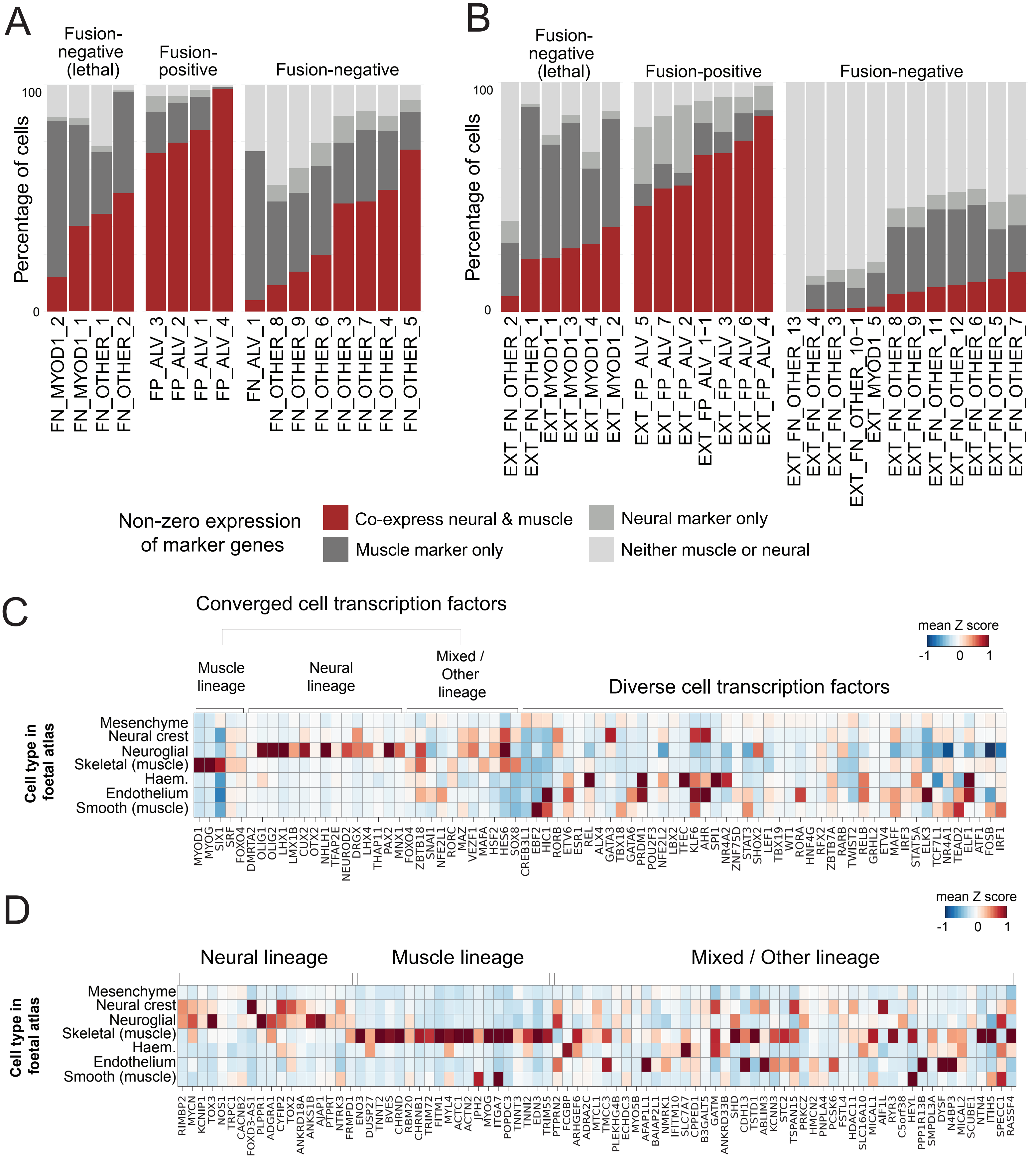

Supplementary Figure 7. Co-expression of converged cell transcription factors from muscle and neural lineages in RMS tumours. (A) Stacked bar chart showing the percentage of single cells that have non-zero expression of at least one: (i) muscle transcription factor, (ii) neural transcription factor, (iii) neither, or (iv) both one muscle and one neural transcription factor. Each bar represents a patient in the single-cell "Discovery" cohort and are facetted based on whether patients are fusion-positive, fusion-negative, or fusion-negative and died of their disease. Transcription factors are listed and annotated as either muscle- or neural-lineage in Supplementary Table 6. **(B)** Stacked bar chart showing the percentage of single nuclei that have non-zero expression of at least one: (i) muscle transcription factor, (ii) neural transcription factor, (iii) neither, or (iv) both one muscle and one neural transcription factor. Each bar represents a patient in the single-nuclei "Extension" cohort and are facetted based on whether patients are fusion-positive, fusion-negative, or fusion-negative and died of their disease. Transcription factors are listed and annotated as either muscle- or neural-lineage in Supplementary Table 6. **(C)** Average expression of converged and diverse transcription factors in different cell types of the foetal developmental spinal atlas. Aggregated expression was calculated using scanpy. **(D)** Average expression of genes from the high-risk transcriptome in different cell types of the foetal developmental spinal atlas. Aggregated expression was calculated using scanpy.
