# Supplementary material for: High-Risk Rhabdomyosarcomas Feature a Convergent Cell State
Source: Cancer Res. Author manuscript; Available in PMC 2026 Oct 10. (PMC13631504; doi:10.1158/0008-5472.CAN-25-4403)
Supplement: Supplementary file 9 [file EMS218232-supplement-Supplementary_Figure_8.pdf]

A

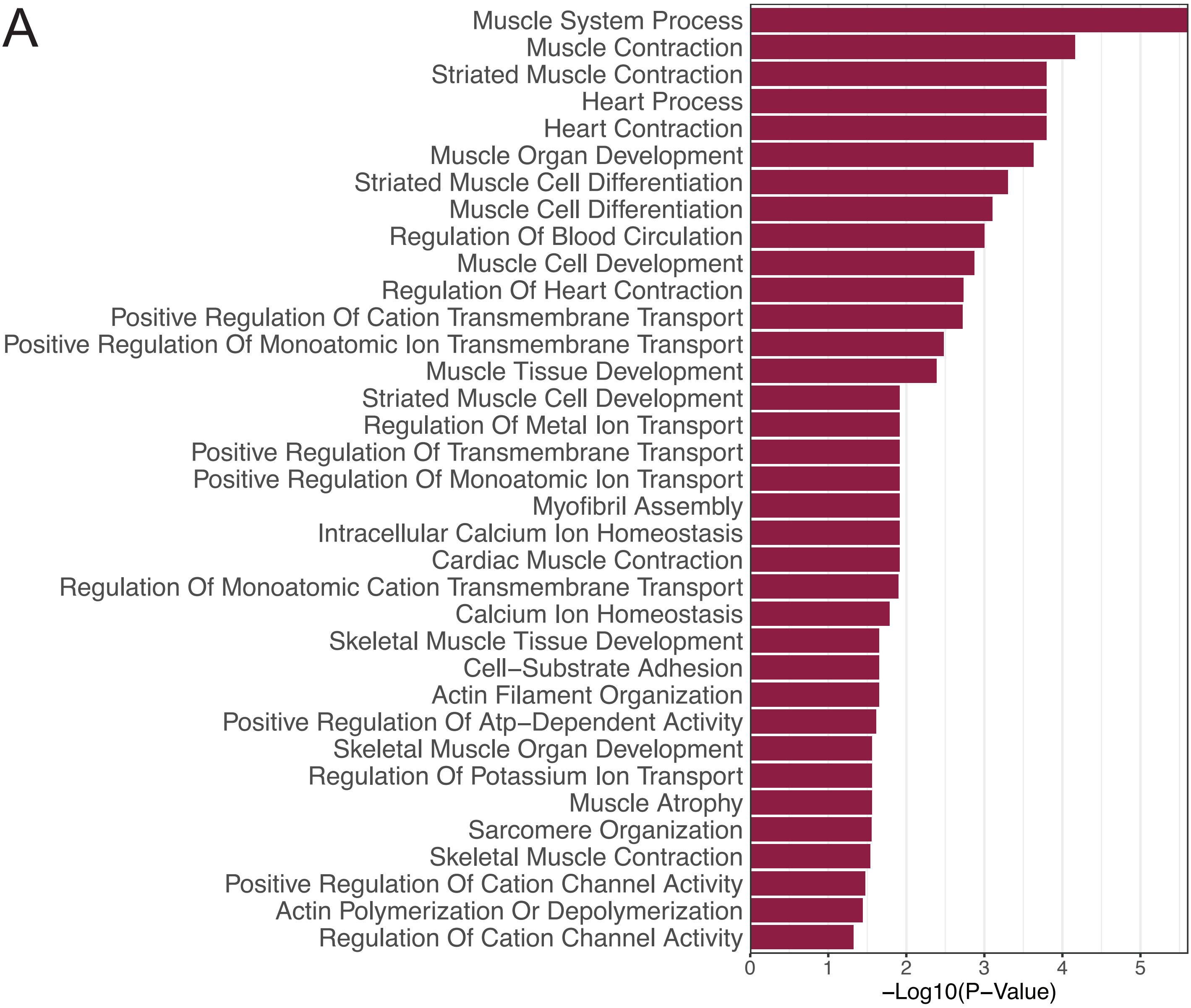

B

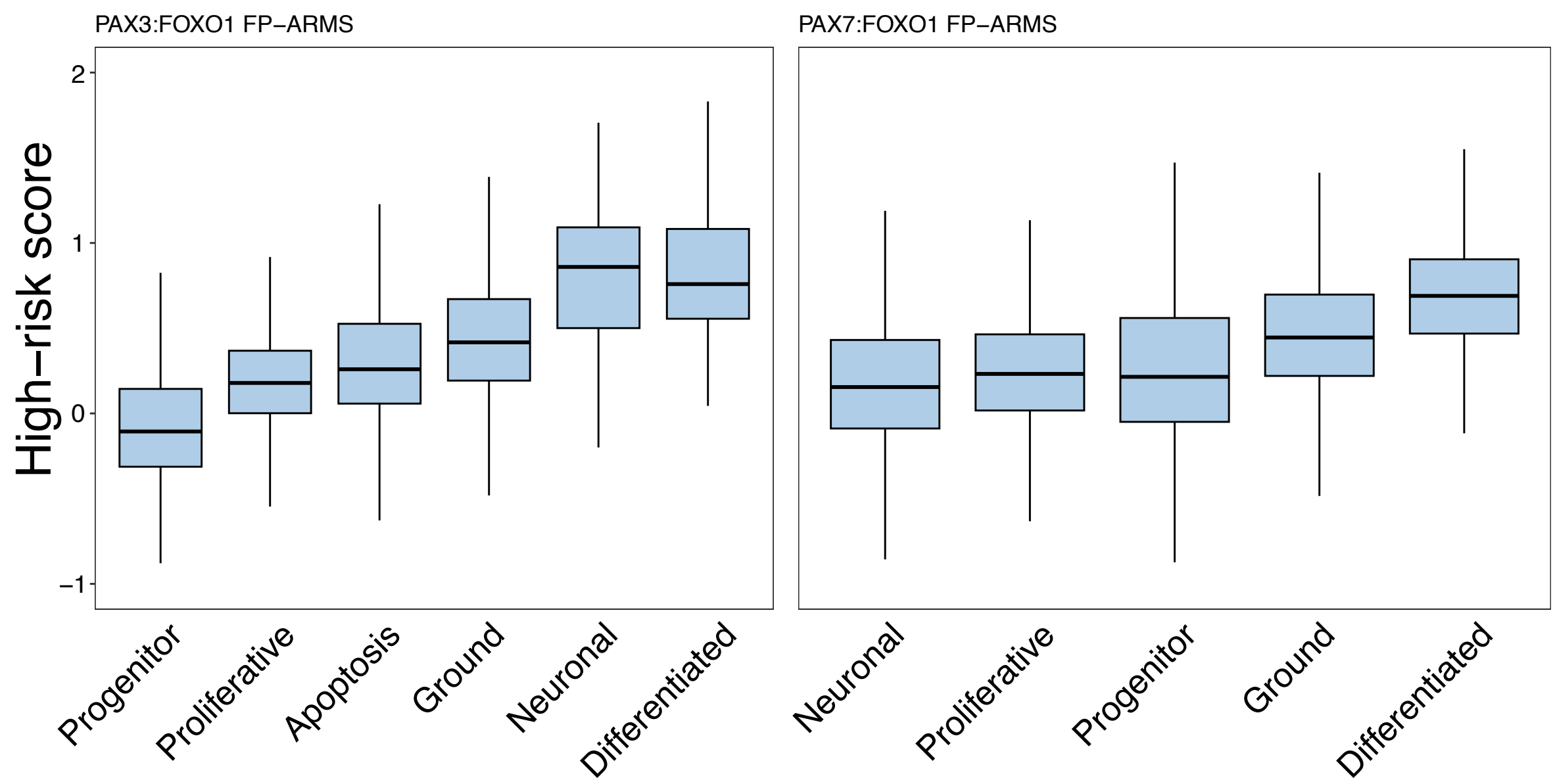

Supplementary Figure 8. Gene Ontology enrichment of the high-risk transcriptome. **(A)** All significant terms from an over-representation analysis of the high-risk transcriptome genes. All "Biological Process" terms from Gene Ontology were tested and the transformed p-values on the x axis are following Benjamini-Hochberg correction. **(B)** Boxplots showing per-cell scores for the high-risk transcriptome across fusion-positive cell populations defined by Danielli et al.14. We show author-defined cell populations in PAX3:FOXO1 FP-ARMS tumours (left) and PAX7:FOXO1 FP-ARMS tumours (right).
