# Supplementary material for: High-Risk Rhabdomyosarcomas Feature a Convergent Cell State
Source: Cancer Res. Author manuscript; Available in PMC 2026 Oct 10. (PMC13631504; doi:10.1158/0008-5472.CAN-25-4403)
Supplement: Supplementary file 10 [file EMS218232-supplement-Supplementary_Figure_9.pdf]

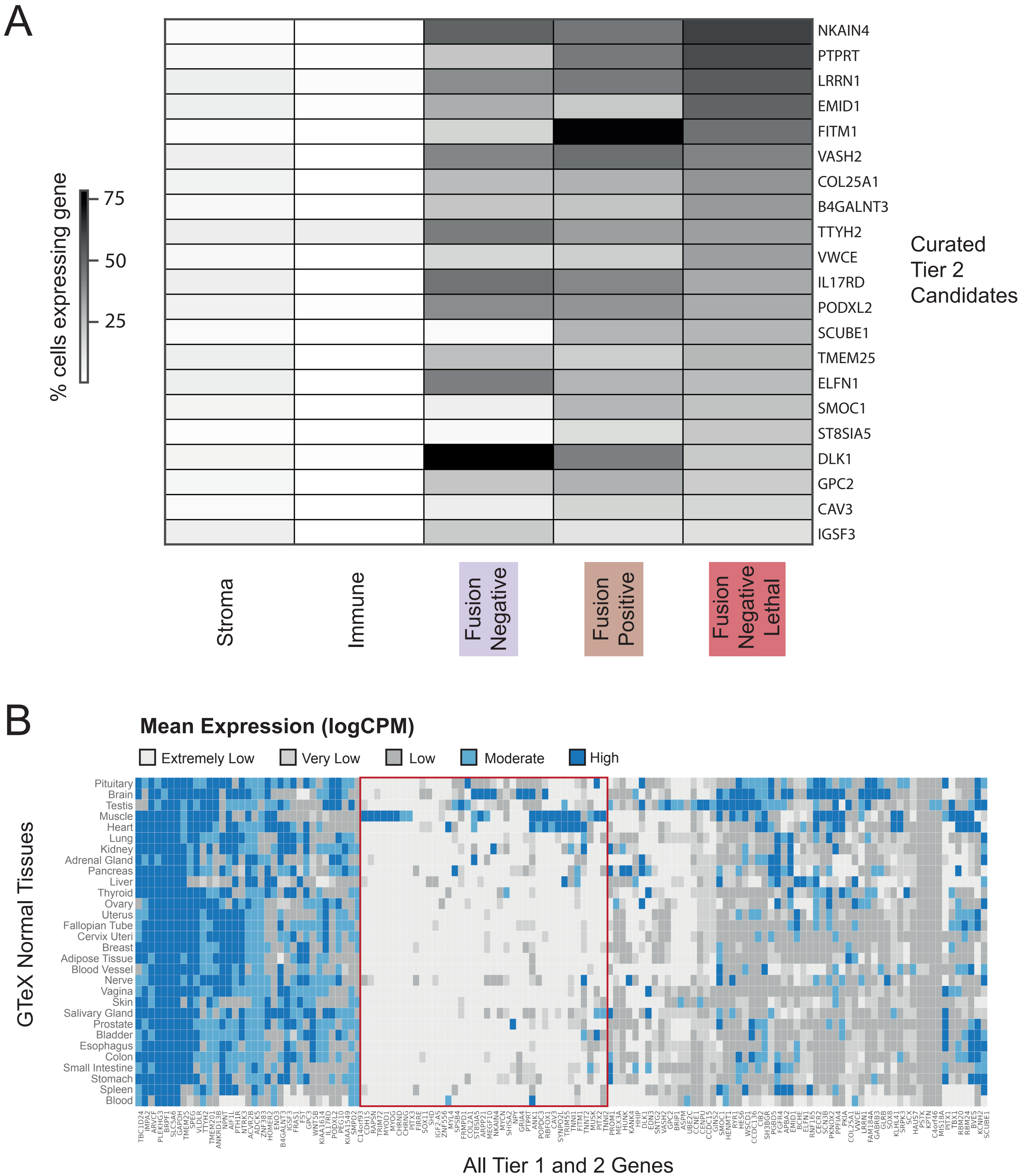

Supplementary Figure 9. Cellular Prevalence and Postnatal Tissue Expression of Prioritized RMS Targets. (A) Bar plot showing the proportion of RMS tumour cells expressing Tier 2 target genes, based on single-cell RNA-seq data. This highlights the cellular prevalence of mid-priority therapeutic candidates within the tumour. **(B)** Heatmap displaying the expression levels of Tier 1 and Tier 2 candidate genes across a panel of normal, postnatal human tissues using GTEx RNA-seq data. Tier 1 genes generally exhibit restricted or absent expression in postnatal tissues, supporting their potential as highly selective therapeutic targets. Tier 2 genes show variable expression, informing risk-benefit considerations for therapeutic development. Together, these analyses support the therapeutic tractability and safety profile of selected foetal-specific targets in RMS.
